# Targeted Genome Mining Reveals the Psychrophilic *Clostridium estertheticum* Complex as a Potential Source for Novel Bacteriocins, Including Cesin A and Estercticin A

**DOI:** 10.3389/fmicb.2021.801467

**Published:** 2022-01-13

**Authors:** Joseph Wambui, Marc J. A. Stevens, Simon Sieber, Nicole Cernela, Vincent Perreten, Roger Stephan

**Affiliations:** ^1^Vetsuisse Faculty, Institute for Food Safety and Hygiene, University of Zurich, Zurich, Switzerland; ^2^Department of Chemistry, University of Zurich, Zurich, Switzerland; ^3^Institute of Veterinary Bacteriology, Vetsuisse Faculty, University of Bern, Bern, Switzerland

**Keywords:** *Clostridium estertheticum*, bacteriocins, lantibiotic, sactipeptide, nisin

## Abstract

Antimicrobial resistance in pathogenic bacteria is considered a major public health issue necessitating the discovery of alternative antimicrobial compounds. In this regard, targeted genome mining in bacteria occupying under-explored ecological niches has the potential to reveal such compounds, including bacteriocins. In this study, we determined the bacteriocin biosynthetic potential of the psychrophilic *Clostridium estertheticum* complex (CEC) through a combination of genome mining and phenotypic screening assays. The genome mining was performed in 40 CEC genomes using antiSMASH. The production of bacteriocin-like compounds was phenotypically validated through agar well (primary screening) and disk diffusion (secondary screening) assays using cell free supernatants (CFS) and partially purified extracts, respectively. Stability of four selected CFS against proteolytic enzymes, temperature and pH was determined while one CFS was analyzed by HRMS and MS/MS to identify potential bacteriocins. Twenty novel bacteriocin biosynthetic gene clusters (BBGC), which were classified into eight (six lantibiotics and two sactipeptides) distinct groups, were discovered in 18 genomes belonging to *C. estertheticum* (*n* = 12), *C. tagluense* (*n* = 3) and genomospecies2 (*n* = 3). Primary screening linked six BBGC with narrow antimicrobial activity against closely related clostridia species. All four preselected CFS retained activity after exposure to different proteolytic, temperature and pH conditions. Secondary screening linked BBGC1 and BBGC7 encoding a lantibiotic and sactipeptide, respectively, with activity against *Bacillus cereus* while lantibiotic-encoding BBGC2 and BBGC3 were linked with activity against *B. cereus*, *Staphylococcus aureus* (methicillin-resistant), *Escherichia coli* and *Pseudomonas aeruginosa*. MS/MS analysis revealed that *C. estertheticum* CF004 produces cesin A, a short natural variant of nisin, and HRMS indicated the production of a novel sactipeptide named estercticin A. Therefore, we have shown the CEC, in particular *C. estertheticum*, is a source of novel and stable bacteriocins that have activities against clinically relevant pathogens.

## Introduction

The *Clostridium estertheticum* complex (CEC) constitutes of 11 spore-forming obligate anaerobic and psychrophilic species, most notably, *C. estertheticum* and *C. tagluense* (Collins, 1992; Spring, 2003; Suetin et al., 2009; Palevich et al., 2021; Wambui et al., 2021a). CEC is mainly associated with a distinct meat spoilage phenomenon colloquially referred to as blown pack spoilage (BPS) that occurs in vacuum packed and refrigerated meat (Wambui and Stephan, 2019). Consistent with their involvement in BPS, CEC have primarily been isolated from animal farmyards and meat processing environment (Esteves et al., 2020, 2021; Wambui et al., 2020d,2021b). Other sources include permafrost (Shcherbakova et al., 2005; Suetin et al., 2009) and Antarctic microbial mat (Spring, 2003). Members of CEC are considered non-pathogenic and do not pose any known food safety risk (Dorn-In et al., 2018), hence they have generally been studied for their role in BPS.

Recently, bioinformatic analyses propagated by whole genome sequencing have identified diverse cryptic genes for synthesis of antimicrobial compounds in different clostridial species (Tracanna et al., 2017; Pahalagedara et al., 2020). Antimicrobial compounds of interest include bacteriocins, which are ribosomally synthesized peptides, that inhibit microbial growth by targeting the cell envelope whereby they can inhibit cell wall synthesis and/or cause pore formation of target cells (Deegan et al., 2006). Bacteriocins are highly diverse with respect to their amino acid sequence, molecular weight, structure, biochemical properties, mode of action and inhibitory spectrum (Cotter et al., 2005). A notable feature of bacteriocins is antimicrobial activity against strains of the same species or strains of closely related species, although, some bacteriocins such as nisin, exhibit antimicrobial activity against strains of distantly related species (Mokoena, 2017). Bacteriocins are classified into two classes, Class I and Class II. Class I bacteriocins, which include lantibiotics and sactipeptides, are small (<5 kDa) and heat-stable post-translationally modified peptides (Cotter et al., 2013). Lantibiotics contain the unusual amino acids lanthionine (Lan), β-methyllanthionine (MeLan), and several dehydrated amino acids (Cotter et al., 2013). Sactipeptides are modified through a thioether bridge linking a cysteine sulfur to α-carbon (Flühe and Marahiel, 2013). Class II bacteriocins are non-modified small (<10 kDa) and heat-stable bacteriocins that are often synthesized as pre-bacteriocins with a N-terminal signal sequence that is cleaved during secretion (Drider et al., 2006).

Bacteriocins are synthesized by a cascade of genes, which are chromosomally or plasmid encoded, and are usually co-localized with genes coding for elements that confer producer strains with self-immunity to their own bacteriocins (Kumariya et al., 2019). As per the recommended nomenclature of lantibiotics, the genes include *lanA*(s) which encode for one or more prepeptides, *lanBC* which encode dehydration and cyclizing proteins in type I lantibiotics and *lanM* which encodes a bifunctional protein in the case of type II lantibiotics, *lanP* which encodes a serine protease that cleaves the leader peptide, *lanT* which encodes a transporter that secretes the lanthipeptide and cleaves the leader peptide in type II lantibiotics, *lanI* which encodes a self-immunity protein, *lanEFG* which encode ABC transporters involved in self-immunity and *lanKR* encoding the regulator and sensor kinase involved in regulation of lanthipeptide synthesis (Arnison et al., 2013). In the case of sactipeptides, the gene clusters are mainly characterized by the structural gene(s) and genes encoding radical SAM proteins (S-adenosylmethionine enzymes containing a typical [4S-4Fe] conserved region) while other common features include the presence of proteases, immunity proteins and transporters (Alvarez-Sieiro et al., 2016). The radical SAM (rSAM) proteins catalyze the formation of thioether bonds in the corepeptide (Chen et al., 2021).

Conventionally, identification of new and active bacteriocins is based on phenotypic screening assays that are often labor- and time-intensive (Cui et al., 2021). Recent advances in genomics and bioinformatics have allowed these challenges to be overcome and provided vast opportunities for genome-mining-based discovery of bacteriocins and other novel natural products (Williams et al., 2020; Zhong et al., 2020). In particular, the conserved domains of the genetic elements in the bacteriocin gene clusters have been exploited using genome mining tools such as BAGEL (Van Heel et al., 2018) and antiSMASH (Blin et al., 2020) leading to discovery of new bacteriocins in microbial groups from diverse niches (Angelopoulou et al., 2020; Mokhtar et al., 2020; Vezina et al., 2020). Despite these advances, the identification rate of bacteriocin gene clusters in *Clostridium* genus was shown to be relatively low in a recent genome mining study (Cebrián et al., 2019). On the other hand, despite occupying an ecological niche that suggests CEC have adopted a different lifestyle compared to the other *Clostridium* species, the bacteriocin biosynthetic potential of CEC is currently unknown. The availability of 50 CEC genomes through recent (Palevich et al., 2021; Wambui et al., 2021a) and other ongoing studies offers a timely opportunity for genome mining aimed at closing this research gap. Therefore, using a targeted approach, we determined bacteriocin production potential of CEC through genome mining. Unexpectedly, this analysis uncovered multiple bacteriocin gene clusters revealing CEC species, and in particular *C. estertheticum*, are a source for bacteriocins. Phenotypic screening to validate bacteriocin production linked partially purified extracts of three preselected CEC strains, which possess four different gene clusters, with antimicrobial activity against clinically relevant pathogens, including *Bacillus cereus*.

## Materials and Methods

### Strains and Growth Conditions

The test and indicator strains used for bacteriocin production and to detect antimicrobial activity, respectively, are shown in Table 1. All strains were previously maintained at –80°C in appropriate media supplemented with 20% glycerol. All strains were reactivated on Columbia Blood Agar supplemented with 5% sheep blood (CBA). CEC strains were incubated anaerobically for 2 weeks at 8°C while *C. gasigenes* and *C. algidicarnis* strains were incubated anaerobically for 72 h at 22°C. Current and subsequent anerobic incubations were carried out in rectangular anerobic boxes (7.0 L; bioMérieux, Inc., Marcy l’Etoile, France) and the anaerobic conditions were generated by three 2.5 L AnaeroGen Sachets (Thermo Fisher Scientific, Massachusetts, United States) per box. The other indicator strains were incubated aerobically for 24 h at 37°C apart from *Bacillus cereus* that was incubated at 30°C. Bacteriocin production by CEC strains was carried out in pre-reduced Reinforced Clostridium Medium (RCM) as specified in subsequent sections. Antimicrobial activity against *Clostridium* spp. and the other indicator strains was tested on CBA and Mueller Hinton agar (MHA), respectively, as described in subsequent sections. For *L. monocytogenes*, MHA was supplemented with 5% sheep blood. Unless stated otherwise, all media and reagents were purchased from Sigma-Aldrich Chemie GmbH, Buchs, Switzerland.

**TABLE 1:** List of strains used in the current study.

Species	Strain id	Status	Source
*C. estertheticum*	CEST001	Test strain	Wambui et al., 2020c
*C. estertheticum*	CF001	Test strain	Wambui et al., 2021a
*C. estertheticum*	CF003	Test strain	Wambui et al., 2021a
*C. estertheticum*	CF004	Test strain	Wambui et al., 2021a
*C. estertheticum*	CF005	Test strain	Wambui et al., 2021a
*C. estertheticum*	CF007	Test strain	Wambui et al., 2021a
*C. estertheticum*	CF008	Test strain	Wambui et al., 2021a
*C. estertheticum*	CF009	Test strain	Wambui et al., 2021a
*C. estertheticum*	CF016	Test strain	Wambui et al., 2021a
*C. estertheticum*	CM034	Test strain	Internal strain collection
*C. estertheticum*	DSM 14864	Test strain	Internal strain collection
*C. tagluense*	CM008	Test strain	Wambui et al., 2021a
*C. tagluense*	CM022	Test strain	Internal strain collection
Genomospecies2	CF011	Test strain	Wambui et al., 2021a
Genomospecies2	CM027	Test strain	Internal strain collection
Genomospecies2	CM028	Test strain	Internal strain collection
*C. estertheticum*	CF010	Indicator strain	Wambui et al., 2021a
*C. tagluense*	CM024	Indicator strain	Wambui et al., 2021a
Genomospecies3	CF012	Indicator strain	Wambui et al., 2021a
*C. lacusfryxellense*	DSM 14205	Indicator strain	Internal strain collection
*C. bowmanii*	DSM 14206	Indicator strain	Internal strain collection
*C. frigoris*	DSM 14204	Indicator strain	Internal strain collection
*C. algidicarnis*	CM003	Indicator strain	Wambui et al., 2020b
*C. gasigenes*	CM005	Indicator strain	Wambui et al., 2021a
*B. cereus*	CH_85	Indicator strain	Internal strain collection
*S. aureus*	MNZ1	Indicator strain	Internal strain collection
*L. monocytogenes*	EGDe	Indicator strain	Internal strain collection
*E. faecalis*	GH24	Indicator strain	Internal strain collection
*E. coli*	ATCC 25922	Indicator strain	Internal strain collection
*P. aeruginosa*	ATCC 27853	Indicator strain	Internal strain collection
*E. cloacae*	NZ 6242-90	Indicator strain	Internal strain collection
*K. pneumoniae*	NZ 513-92	Indicator strain	Internal strain collection

### *Clostridium estertheticum* Complex Genomes

Ten publicly available whole genome sequences of CEC as of December 2020 were downloaded from the NCBI database. Additional 40 genomes from Wambui et al. (2021a) and ongoing studies were included in the analysis. The genomes are listed in Supplementary Table 1. To avoid overestimation of gene clusters, genomes from nearly clonal strains, were represented by only one genome. In total, 40 CEC genomes were included in the genome mining analysis.

### *In silico* Prediction and Characterization of Bacteriocin Biosynthetic Gene Clusters

Cryptic bacteriocin gene clusters were predicted using antiSMASH v.6 (Blin et al., 2020) and further validated using BAGEL v.4 (Van Heel et al., 2018). The amino acid sequences of corepeptides within the clusters were predicted and downloaded from the antiSMASH webserver. The amino acid sequences of 41 corepeptides of known lantibiotics were also downloaded from BACTIBASE webserver in April 2021 (Hammami et al., 2010). The list was supplemented with other corepeptides of other known lantibiotics through a literature search. A similar search strategy was used to identify seven corepeptide sequences of currently known sactipeptides. The sequences were aligned using CLC Workbench Genomics v. 8.1 (Qiagen, Aarhus, Denmark), and phylogenetic trees created from the aligned sequences in the CLC Workbench Genomics using the Maximum likelihood Phylogeny method. Bootstraps were based on 1,000 replicates. The phylogenetic grouping of bacteriocin within CEC was based on the *rpoB* phylogeny. Briefly, the *rpoB* gene sequences of the 40 CEC were identified using blastn and extracted with samtools using the faidx option and the blast results as input (Altschul et al., 1990; Li et al., 2009). The sequences were aligned and the *rpoB* tree phylogenetic created in CLC Workbench Genomics.

### Nanopore Sequencing and Plasmid Characterization

To fully map the identified bacteriocin gene clusters and determine whether they were localized within the chromosomal or plasmid DNA, 16 in-house CEC strains (Supplementary Table 2) were completely sequenced using MinION Oxford Nanopore Technology (ONT) (Oxford Nanopore Technologies, United Kingdom). Genomic DNA was extracted using the MasterPure Complete DNA and RNA Purification Kit (Lucigen LubioScience, Zürich, Switzerland) as per manufacturer’s instructions. ONT library was prepared using the 1D ligation sequencing kit (SQK-LSK109) and the native barcoding expansion kit (EXP-NBD104), and sequenced with MinION MK1b device using a R9.4.1 SpotON flow cell (ONT). The ONT reads were base-called and demultiplexed using Guppy software (v4.4.1). Trimming and size end filtering were carried out using Cutadapt v2.5. Illumina sequencing was performed-inhouse as previously described by Wambui et al. (2021a) without modification. The genome sequences were assembled *de novo* and circularized using Unicycler v0.4.8 run with default parameters, using the paired-end Illumina reads and ONT reads larger than 10 kb (Wick et al., 2017). The sequences were annotated using RAST annotation webserver (Brettin et al., 2015). The plasmids encoding the BBGC were visualized with SnapGene viewer v.2^1^ and InkSpace v.1.02^2^ softwares.

### Primary Screening for Antimicrobial Screening

The 16 in-house CEC test strains with cryptic bacteriocin biosynthetic clusters were grown anaerobically in 30 ml RCM (each in 3 × 10 ml) in Hungate Anaerobic Tubes (BellCo Glass Inc., New Jersey, United States) at 8°C for 4 weeks without agitation. Attempts to grow one of the strains, *C. estertheticum* CF016, were unsuccessful. After incubation, successfully grown cultures of each strain were pooled together and cell free supernatants (CFS) were prepared by centrifugation at 8,000 × g for 15 min at 4°C and subsequent filter sterilization using 0.22 μm filter membranes (Thermo Fisher Scientific, Massachusetts, United States). The CFS were aliquoted and stored at –80°C. The antimicrobial activity of the CFS was tested using agar well diffusion assay (AWDA). Indicator strains listed in Table 1 were standardized to McFarland 0.5 using sterile 0.9 NaCl solution then spread on respective media using sterilized swabs. At most, five wells measuring eight millimeters were made in each solid media using a sterile corkborer. CFS (90 μl) was pipetted into respective wells and the indicator strains incubated under respective growth conditions. The antimicrobial activity was determined as presence of inhibition of zones, which were measured to the nearest millimeters using Vernier calipers. Three biological replicates were performed.

### Effects of Enzyme, pH, Temperature on Antibacterial Activity

The effect of proteolytic enzymes, pH and temperature on antimicrobial activity was carried out for four CFS from the four representative strains *C. estertheticum* CF004, genomospecies2 CF011, *C. estertheticum* CF003 and *C. tagluense* CM008. The sensitivity to 1 mg/ml proteolytic enzymes; trypsin (pH 7.2), pepsin (pH 2.0), protease (pH 7.2), and proteinase K (pH 7.2), was determined by incubating the CFS for 2 h at 37°C. The enzymes were inactivated at 90°C for 3 min. The pH stability of the CFS was determined by adjusting the pH to 2, 4, 8, and 10 using 1M HCl or NaOH. The CFS was incubated at respective pH for 3 h at 37°C, then the pH was readjusted to 6.0. The temperature stability of the BLIS was determined by incubating the CFS at 60, 80°C for 1 h, 100°C for 30 and 121°C for 15 min. The residue activity of treated CFS (90 μl) was determined against untreated CFS (90 μl). *C. bowmanii* or *C. lacusfryxallense* (Table 1) were used as indicator strains in AWDA as described above. Treated and non-treated nisin (12.5 ppm; 90 μl) were used as controls. The residual activity was recorded as the difference between the inhibition zone of treated and non-treated samples. All experiments were carried out in three biological replicates.

### Secondary Screening for Antimicrobial Activity

For the secondary screening assay, three strains, *C. estertheticum* CF004, *C. tagluense* CM008 and *C. estertheticum* CF009 carrying BBGC1 (and BBGC7), BBGC2 and BBGC3, respectively, were selected for cultivation. Antimicrobial compounds were partially purified in a three-stage process as previously described (O’Sullivan et al., 2020) with slight modifications. Briefly, the strains were grown anaerobically in 500 ml RCM (6 weeks; 8°C; no agitation). Cell free supernatant was harvested by centrifugation (8,000 × g, 15 min, 4°C) followed by filter sterilization through 0.2 μm filter membranes. XAD-16N resin (50 g) was added to the CFS and gently shaken for 24 h (4°C). The resin was washed with 250 ml distilled water then antimicrobials eluted with 150 ml IPA (80% propan-2-ol, 0.1% trifluoroacetic acid). The IPA was removed with a rotary evaporator (Heidolph Instruments GmbH & Co., Schwabach, Germany) at 45°C until the volume was 80–100 ml then the pH adjusted to 4.5 with 1N NaOH. The samples were applied to Econo-Columns (Bio-Rad Laboratories, Inc., California, United States) containing 40 ml SP Sepharose beads. The columns were preequilibrated with 50 mM sodium acetate buffer (pH 4.5). Each column was washed with 50 ml of the buffer and the antimicrobials eluted with 100 ml of the buffer containing 1 M NaCl. The eluents were applied to 5 g × 20 ml C_18_ solid-phase extraction (SPE) columns preequilibrated with methanol and water. Each column was washed with 20 ml distilled water and the antimicrobials eluted with 20 ml IPA. The samples were dried to completeness by rotary evaporation, resuspended in 1 ml methanol and stored at –80°C for later use. Antimicrobial activity of the compounds was determined using disk diffusion assay as previously described (Wambui et al., 2020a) with slight modifications. Briefly, 6 mm disks (Whatman plc) were impregnated with 50 μl of the compounds and dried under a laminar flow cabinet (3 h; room temperature). Indicator strains *B. cereus*, Methicillin-resistant *S. aureus*, *P. aeruginosa* and *E. coli* (Table 1), were standardized to 0.5 McFarland and spread on MHA and the dried disks affixed on top. The plates were initially left to stand (3 h; room temperature) to allow the compounds to diffuse then incubated at 37°C for 18 h. *B. cereus* was incubated at 30°C. The antimicrobial activity, carried out in three biological replicates, was determined as presence of clear zones around the disks.

### Isolation Method, HRMS and MS/MS Analysis of BBGC1 and BBGC7 Encoded Bacteriocins

*C. estertheticum* CF004 carrying BBGC1 and BBGC7 encoded lantibiotic and sactipeptide, respectively, was selected to validate bacteriocin production in CEC. The strain was grown in 2,000 ml RCM as described above. Purification steps were also as described above with slight modifications. In the first step, 200 g of XAD-16N, 1 L wash water and 600 ml IPA were used. Part of the sample (1 μl injection) was analyzed by HRMS and MS/MS with a Vanquish™ Horizon UHPLC System (Thermo Fisher Scientific, Waltham, United States) connected to a Vanquish eλ detector and a timsTOF Pro TIMS-QTOF high-resolution mass spectrometer (Bruker Daltonics, Bremen, Germany). Separation was performed with an Acquity BEH C18 HPLC column (1.7 μm particle size, 2 × 100 mm, Waters) kept at 30°C. The mobile phase consisted of A: H_2_O + 0.1% HCOOH and B: CH_3_CN + 0.1% HCOOH. A linear gradient was isocratic at 3% B for 0.5 min and run from 3 to 100% B within 8.5 min followed by flushing with 98% B for 3 min at a flow rate of 450 μl min^–1^. The mass timsTOF Pro spectrometer was operated in the positive electrospray ionization mode at 4’000 V (–4,000 V) capillary voltage and –500 V (500 V) endplate offset with a N_2_ nebulizer pressure of 2.8 bar and dry gas flow of 8 L min–1 at 220°C. Spectra were acquired in the mass range from m/z 50 to 2,000 at 20,000 resolution (m/z 500 full width at half maximum) and at a 1.5 Hz rate. The mass analyzer was calibrated at the beginning of each LC run between m/z 158 and 1’450 using a 10 mM solution of sodium formate that was injected using a 6- port-valve with a 20 μl loop at a resolution of ca. 25,000 (m/z 622) giving a mass accuracy below 2 ppm. MS/MS spectra were acquired at a collision energy of e.g., 35 eV with an 3 m/z isolation width in a mass range from m/z 50 to 600 at a 2.0 Hz rate. N2 was used as a collision gas.

### Data Analysis

Zones of inhibitions for the antimicrobial activity were described as means and standard deviation. Differences in means (*p* ≤ 0.05) after exposure of CFS to different proteolytic enzymes, temperature and pH were determined using ANOVA. The data analyses were carried out in R Studio Version 1.1.463 (RStudio, Inc., Boston, United States).

## Results

### Evidence of Plasmid Encoded Bacteriocin Biosynthetic Gene Clusters Within *Clostridium estertheticum* Complex

**FIGURE 1:**
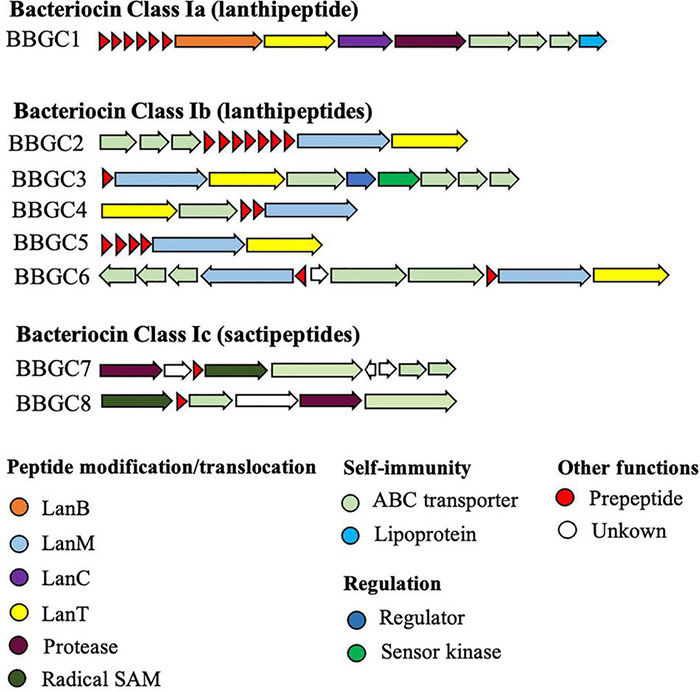
Bacteriocin biosynthetic gene clusters identified within *Clostridium estertheticum* complex (CEC). The eight clusters represent 20 clusters identified in 18 CEC genomes. The gene clusters were not drawn to scale.

We analyzed 40 CEC genomes for the presence of bacteriocin biosynthetic gene clusters (BBGC). We uncovered 20 BBGC in 18 out of the 40 genomes (Table 2). The BBGC were broadly classified into lantibiotics (*n* = 18) and sactipeptides (*n* = 2). Based on gene composition and organization, the clusters were grouped into eight distinct gene clusters (Figure 1), which are herein referred to as bacteriocin biosynthetic gene clusters 1 to 8 (BBGC1 to BBGC8). Slight variations existed in BBGC1 and BBGC2 hence the clusters from respective genomes are shown in Supplementary Figure 1. The two clusters were also the most prevalent having been identified in six and seven genomes, respectively. The genome of *C. estertheticum* CF004 had a lantibiotic (BBGC2) and a sactipeptide (BBGC7) cluster while the genome of *C. estertheticum* CF016 had two different lantibiotic clusters (BBGC2 and BBGC6). The other 16 genomes had one BBGC each.

**TABLE 2:** Characteristics of bacteriocin biosynthetic gene clusters (BBGC) in *Clostridium estertheticum* complex genomes predicted *in silico* using antiSMASH webserver.

Species	Strain	Class	Sequence of core peptide(s)*	Cluster	Proposed name^†^
*C. estertheticum*	CEST001	Ia	ITSWSLCTAGCITGRIMGCNK	BBGC1	Cesin A
*C. estertheticum*	CM034	Ia	ITSWSLCTAGCITGRIMGCNK	BBGC1	Cesin A
*C. estertheticum*	CF001	Ia	ITSWSLCTAGCITGRIMGCNK	BBGC1	Cesin A
*C. estertheticum*	CF004	Ia	ITSWSLCTAGCITGRIMGCNK	BBGC1	Cesin A
*C. estertheticum*	CF007	Ia	ITSWSLCTAGCITGRIMGCNK	BBGC1	Cesin A
*C. estertheticum*	CF008	Ia	ITSWSLCTAGCITGRIMGCNK	BBGC1	Cesin A
*C. estertheticum*	DSM 14864	Ib	GGGVITTFTHECYYNSVSPASWGGCCK	BBGC2	Estercin A-E
*C. estertheticum*	MA19	Ib	GGGVITTFTHECYYNSVSPASWGGCCK	BBGC2	Estercin A-E
*C. estertheticum*	CF016	Ib	GGGVITTFTHECYYNSVSPASWGGCCK	BBGC2	Estercin A-E
*C. tagluense*	CM008	Ib	GGGVITTFTHECYYNSVSPASWGGCCK GGGVINTFTHECYYNSVSPASWGGCCK	BBGC2	Estercin A-T
Genomospecies2	CF011	Ib	GNGVITTFTHECYYNSVSPASWGGCCK	BBGC2	Estercin A-G
Genomospecies2	CM027	Ib	GNGVITTFTHECYYNSVSPASWGGCCK	BBGC2	Estercin A-G
Genomospecies2	CM028	Ib	GNGVITTFTHECYYNSVSPASWGGCCK	BBGC2	Estercin A-G
*C. estertheticum*	CF003	Ib	ASSGAICTATTECTYFSAICC	BBGC3	Estercin B
*C. estertheticum*	CF009	Ib	ASSGAICTATTECTYFSAICC	BBGC3	Estercin B
*C. estertheticum*	CF005	Ib	TISTHLCATVYLSAVSIAGSIKLTKWILN TTLPCSVLWTVYTAAVSTACCAAATVGASLMISGQIFN	BBGC4	Estercin C
*C. tagluense*	FP2	Ib	TNDWVSKQAANIAGLGTNYGRVCTVSAECDFTNLCAGTK TNDWVSKQAANIAGLGTNYGRVCTVSAECDFTNLCGGTK TNDWLSKKAANMAGLGNNYGRVCTVSAECDFTNLCGDTK TNDWLSKKAANMAGLGNNYGRICSVSAECEGFLCGDTK	BBGC5	Taglucin A
*C. estertheticum*	CF016	Ib	GGTTPVVSAISATVASATAVSALFTVTSACTKSCNK GWVSTILQGTVGCLASYALGNQGKICTWSVECQNNC	BBGC6	Estercin D
*C. estertheticum*	CF004	Ic	ELNCWACLGCVGCAACVFTLALASAMSALTGSNR	BBGC7	Estercticin A
*C. tagluense*	CM022	Ic	FNCACICYKESQISETKSGSWAPFTGGCYYSCDSAIAGNLTGNKNTSHSA	BBGC8	Taglucticin A

*The proposed name of each encoded bacteriocin is given.*

*Predicted using antiSMASH in the case of lantibiotics or sequence alignment against other known sactipeptides.

*^†^The suffix “-X” denotes species specific variant e.g., Estercin A–E denotes Estercin A variant identified in C. estertheticum.*

**FIGURE 2:**
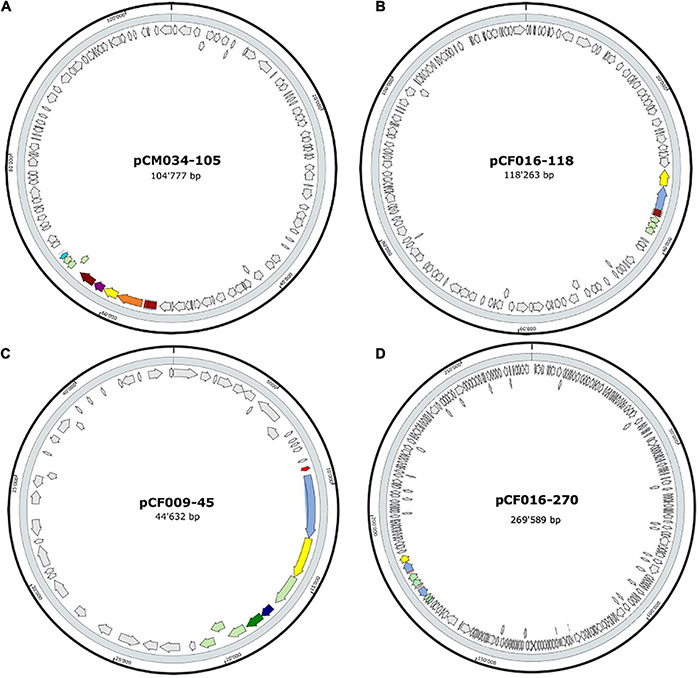
Localization of bacteriocin (lantibiotics) biosynthetic gene clusters (BBGC) in the plasmids of *Clostridium estertheticum* strains. **(A)** Localization of Class Ia lantibiotic BBGC1 in *C. estertheticum* CM034. **(B–D)** Localization of Class Ib lantibiotic BBGC2, BBGC3 and BBGC6 in *C. estertheticum* strains CF016, CF009 and CF016, respectively.

**FIGURE 3:**
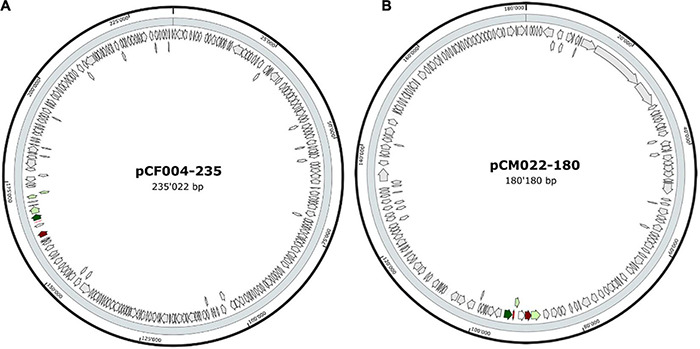
Plasmid localization of bacteriocin (sactipeptides) biosynthetic gene clusters (BBGC). **(A)** Localization of sactipeptide BBGC7 in *C. estertheticum* CF004. **(B)** Localization of sactipeptide BBGC8 in *C. tagluense* CM022.

The 20 BBGC were encoded in plasmids. Each of the two BBGC in *C. estertheticum* strains CF004 and CF016 were encoded on different plasmids. The bacteriocin encoding plasmids were of varying sizes. BBGC1 and BBGC2 were encoded on plasmids whose size ranged from 57 to 105 kbp (Figure 2A and Supplementary Figure 2) and 68–118 kbp (Figure 2B and Supplementary Figure 3), respectively. Both BBGC3 clusters were encoded on 45 kbp plasmids (Figure 2C and Supplementary Figure 4). BBGC4 was encoded on the smallest plasmid (25 kbp; Supplementary Figure 4) while BBGC6 was encoded on the largest plasmid (270 kb; Figure 2D). Both sactipeptides BBGC7 and BBGC8 were encoded on 180 kbp and 235 kbp mega-plasmids, respectively (Figure 3). Taken together, the CEC bacteriocins are most frequently encoded in plasmids.

### Description of Bacteriocin Biosynthetic Gene Clusters

#### Type I Lantibiotic Biosynthetic Gene Cluster

A more detailed look at the bacteriocin clusters revealed BBGC1 comprised *lanB* and *lanC*, which are signature biosynthetic genes for Class Ia bacteriocin (Type I lantibiotic), and encode dehydratase and cyclase proteins of the LanB and LanC family domain proteins, respectively (Figure 1). In this cluster, six to eight prepeptide genes were present (Supplementary Figure 1). The genes encoded 21 aa identical corepeptides (Table 2). Other genes that characterize lantibiotic type I biosynthetic gene clusters were present downstream the structural peptide, including ATP-binding cassette (ABC) transporter, peptidase, and self-immunity proteins (lantibiotic export ABC transporters and lantibiotic immunity protein).

#### Type II Lantibiotic Biosynthetic Gene Clusters

Six different Class Ib bacteriocin (Type II lantibiotics) biosynthetic gene clusters were identified and were all characterized by genes encoding the multifunctional biosynthetic proteins of the LanM family domain (Figure 1). Other genes encoding LanT family domain proteins and ABC transporters proteins were also identified. Regulatory proteins, transcriptional regulator and sensor kinase, were identified in BBGC3. The number of prepeptide genes varied from one in BBGC3 to seven in BBGC2 (Table 2). Despite encoding the highest number of prepeptides (*n* = 6–7), the predicted corepeptides of the three genomospecies2 genomes were identical. Four prepeptides were identified in *C. tagluense* CM008, one of which had one amino acid substitution resulting in two variants of the corepeptide (Table 2). The variant with the amino acid substitution was 100% identical to the corepeptides in genomospecies2 clusters. Overall, three variants of lantibiotic from BBGC2, whose sequence identity was 92.59%, were predicted in three CEC species. In BBGC4, BBGC5 and BBGC6, two, four, and two prepeptides were identified. The sequence identity of predicted BBGC4, BBGC5 and BBGC6 corepeptides were 16.28, 69.23, and 15.79%, respectively.

#### Sactipeptide Biosynthetic Gene Clusters

The two sactipeptide clusters were characterized by genes encoding the radical SAM family domain (Figure 1). Other genes encoding ABC transporters proteins and proteases were also identified. In addition, up to three genes encoding proteins of unknown function were present. Each cluster had one prepeptide gene.

### Phylogenetic Grouping of Bacteriocin Biosynthetic Gene Clusters

**FIGURE 4:**
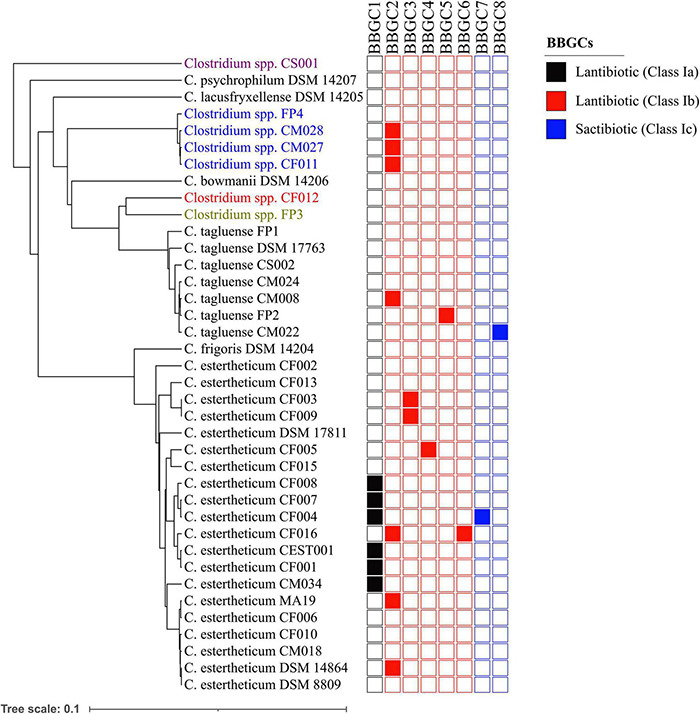
Distribution of bacteriocin biosynthetic gene clusters (BBGCs) within *Clostridium estertheticum* complex. The strains with olive, blue, red and purple fonts belong to genomospecies1, genomospecies2, genomospecies3, and genomospecies4, respectively. The phylogenetic tree is based on the *rpoB* gene. The bar indicates 0.1 substitutions per nucleotide position.

Plasmids are mobile genetic elements and therefore, the distribution of bacteriocin biosynthetic gene clusters within CEC were determined (Figure 4). BBGC2 was the most widespread cluster having been identified in three *C. estertheticum*, three genomospecies2 and one *C. tagluense* genomes. Within genomospecies2, the three genomes carrying the BBGC2 formed a subgroup while within *C. estertheticum*, only two genomes of DSM 14864 and MA19 formed a monophyletic subgroup. Surprisingly, *C. estertheticum* CF016, carrying the BBGC2 and BBGC6, formed a subgroup with five genomes of *C. estertheticum* CEST001, CF001, CF004, CF007, and CF008, all of which harbored the BBGC1 (and BBGC7 in the case of *C. estertheticum* CF004). With eight clusters, this was the most bacteriocin-dense monophyletic subgroup. However, *C. estertheticum* CM034 carrying BBGC1 was outside this subgroup. The two *C. estertheticum* genomes CF003 and CF009 carrying BBGC3 also formed a subgroup. Similar observations were made for the three *C. tagluense* genomes CM008, FP2 and CM022 carrying BBGC2, BBGC5, and BBGC8, respectively. Conclusively, majority of the plasmid-encoded bacteriocin gene clusters were distributed in strains independent of their position in the phylogenetic tree, suggesting strongly horizontal transfer of the clusters.

### Phylogenetic and Sequence Relatedness of *Clostridium estertheticum* Complex Bacteriocins to Known Bacteriocins

**FIGURE 5:**
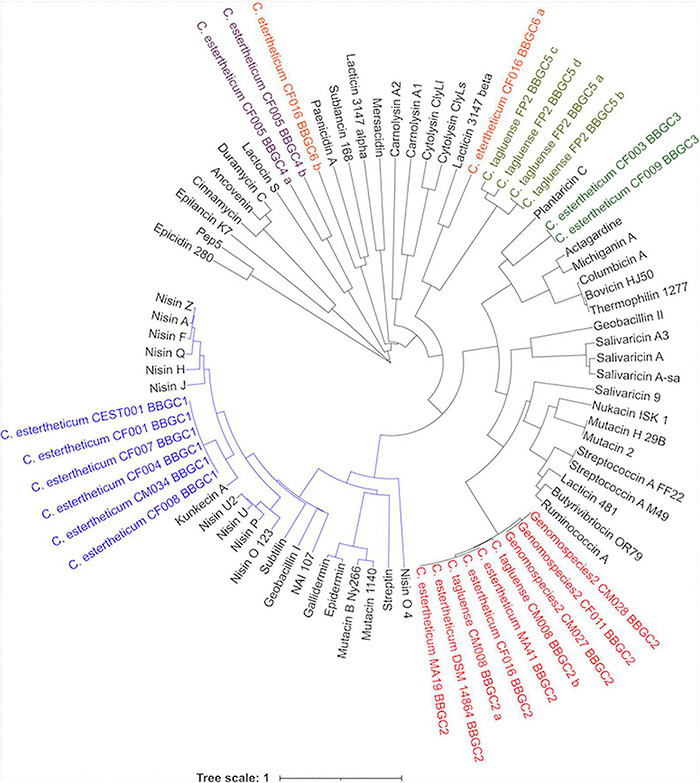
Phylogenetic relatedness of *Clostridium estertheticum* complex lantibiotics and known lantibiotics. The BBGC1 lanthipeptides (blue font) clustered with 11 known natural variants of nisin. The blue node denotes prominent members of class I lanthipeptides. Gene clusters for Class II lanthipeptides BBGC2, BBGC3, BBGC4, and BBGC5 and BBGC6 are indicated in red, green, purple olive and orange fonts, respectively. The bar indicates 1 substitution per nucleotide position.

Next, the phylogenetic relatedness of CEC lantibiotics to known lantibiotics was studied (Figure 5). All BBGC1 clusters encode the same lantibiotic that clustered with 11 known natural variants of nisin, suggesting it is a novel nisin variant. Sequence analysis showed the BBGC1 encoded variant is the shortest (21 aa) among known natural variants of nisin. Three amino acid residues, Trp4, Ala9, and Ile16 were unique to the BBGC1 lantibiotic (Supplementary Figure 5) while its sequence identity with nisin A was 44.1%. The BBGC1 lantibiotic is herein named cesin A (denoting *C. estertheticum* inhibitory substance), due to its phylogenetic relatedness to the nisin variants. The three variants of BBGC2 lantibiotic clustered with nine lantibiotics, but the sequence identity was highest with salivaricin 9 (37.3%). The lantibiotics from BBGC3 clustered with plantaricin C and the sequence identity was 42.3%. The BBGC4 lantibiotic was distantly related to lactocin S (2.3%). BBGC5 lantibiotic did not cluster with any known lantibiotic. The BBGC6 lantibiotic peptides were lowly identical to lacticin 3147 beta (33.3%) and paenicidin A (18.2%). The phylogenetic relatedness of CEC sactipeptides to known sactipeptides is shown in Supplementary Figure 6. The BBGC7 was lowly identical to thuricin H (28.6%) while the BBGC8 sactipeptide did not cluster with any of the known sactipeptides. Given the encoded CEC bacteriocins showed low sequence identity to known bacteriocins, we propose they are novel members of their respective classes. To facilitate future reference and characterization, the names of bacteriocins encoded by each cluster have been proposed in Table 1.

### Primary and Secondary Screening for Antimicrobial Activity

**FIGURE 6:**
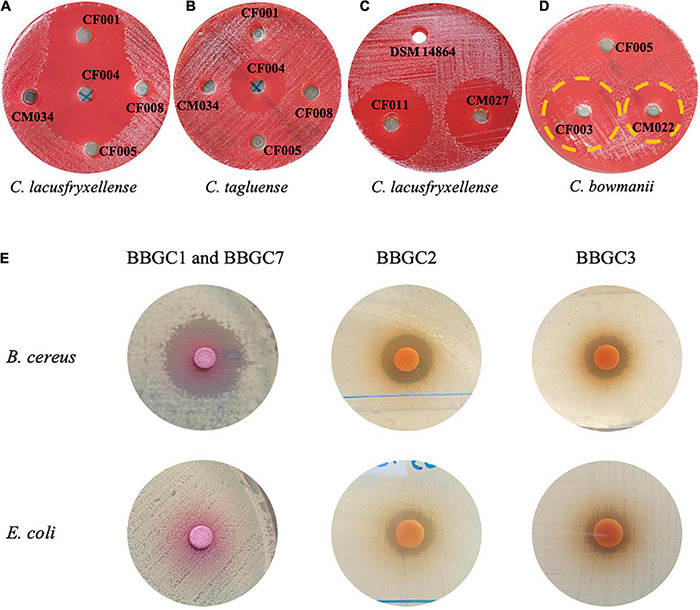
Antimicrobial activity of cell free supernatants **(A–D)** and partially purified extracts **(E)** of selected *Clostridium estertheticum* complex strains possessing bacteriocin biosynthetic gene clusters (BBGC). **(A)** *C. estertheticum* strains CF001 (BBGC1) and CF004 (BBGC1 and BBGC7) were active against *C. lacusfryxellense*. **(B)** *C. estertheticum* CF004 (BBGC1 and BBGC7) was active against *C. tagluense.* **(C)** Genomospecies2 strains CF011 (BBGC2) and CM027 (BBGC2) were active against *C. lacusfryxellense* **(D)**. *C estertheticum* CF003 (BBGC3) and (BBGC) *C. tagluense* CM022 (BBGC8) were active against C. bowmanii. **(E)** Partially purified extracts of *C. estertheticum* CF004 (BBGC1 and BBGC7) was active against *B. cereus* while extracts of *C. tagluense* (CM008) BBGC2 and *C. estertheticum* CF009 (BBGC3), respectively, were active against *B. cereus* and *E. coli*.

To determine the expression of the bacteriocin gene clusters, the antimicrobial activity of the strains with a BBGC was tested against a wide range of test strains. CFS of three strains that exclusively harbor BBGC1 inhibited six out of eight strains tested psychrophilic and psychrotrophic clostridia strains (PPC). Despite carrying BBGC1, no activity was observed for *C. estertheticum* CF008 and CM034 (Supplementary Table 3 and Figures 6A,B). Interestingly, CFS from *C. estertheticum* CF004 carrying BBGC1 and BBGC7 inhibited all eight PPC and exclusively inhibited *C. tagluense* CM024 (Supplementary Table 3 and Figure 6B). BBGC2 also corresponded with broader antimicrobial activity that was observed against all five out of eight tested PPC, but this activity was only observed in the CFS of genomospecies2 strains (Supplementary Table 4 and Figure 6C). The CFS of both strains carrying BBGC3 were active against *C. bowmanii* DSM 14206 and *C. frigoris* DSM 14204 while the CFS of BBGC8 carrying strain was active against *C. lacusfryxellense* DSM 14205, *C. bowmanii* DSM 14206 and *C. frigoris* DSM 14204 (Supplementary Table 4 and Figure 6D). No activity was observed against non-clostridia strains during the primary screening. However, the secondary screening assay using partially purified bacteriocins from strains carrying BBGC1 (and BBGC7), BBGC2 and BBGC3 showed broader antimicrobial activity (Figure 6E). Specifically, the partially purified extract of *C. estertheticum* CF004 carrying BBGC1 and BBGC7 was active against *B. cereus* but not against *E. coli*. This was in contrast to the partially purified extracts of *C. tagluense* CM008 and *C. estertheticum* CF009 carrying BBGC2 and BBGC3, respectively, which were active against both *B. cereus* and *E. coli*. Further screening showed the two extracts were active against methicillin-resistant *S. aureus* and *P. aeruginosa* (Supplementary Figure 7). Taken together, the data suggest the majority of the CEC bacteriocins are expressed, however, broad antimicrobial activity is only evident after concentration of the compounds.

### Stability of Selected Antimicrobial Compounds Against Proteolytic Enzymes, Temperature, and pH

**FIGURE 7:**
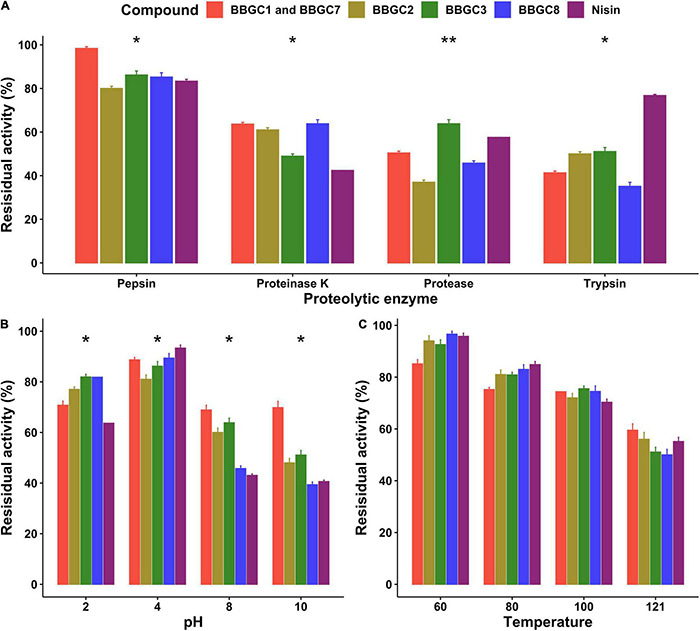
Effects of proteolytic enzymes, pH and temperature on antimicrobial activity of cell free supernatants (CFS) of four *Clostridium estertheticum* complex strains carrying bacteriocin biosynthetic gene clusters. **(A)** Residual activity of the CFS after treatment with 1 mg/ml proteolytic enzymes at 37°C for 2 h. **(B)** Residual activity of the CFS after exposure to acidic (pH 2 and 4) and alkaline condition (pH 8 and 10) at 37°C for 3 h. **(C)** Residual activity of the CFS after exposure to 60 and 80°C from 1 h, 100°C for 30 min and 121°C for 15 min. All tests were performed in triplicate, and data are shown as mean values ± SD. The means were compared for each treatment using ANOVA (*p* = 0.05). Nisin (12.5 ppm) was used as control. *p < 0.05; **p < 0.01.

The stability of the active bacteriocin-like compounds, the residual activity of CFS of strain carrying BBGC1 (and BBGC7), BBGC2, BBGC3, and BBGC8 was studied (Figure 7). The residual activity was highest after treatment with pepsin with CFS of BBGC1 and BBGC7 retaining the highest activity. There were significant decreases in activity after treatment with proteinase K, protease and trypsin although BBGC1 (and BBGC7), BBGC2, and BBGC8 retained higher activity after proteinase K treatment. After treatment with protease, BBGC3 retained the highest activity while all CFS showed less residual activity after treatment with trypsin compared to nisin. All CFS and nisin showed highest residual activity at pH 2 and pH 4. However, all four CFS were more active than nisin at pH 2. Although an overall decrease in the residual activity of all compounds was observed at pH 8 and pH 10, the CFS of BBGC1 (and BBGC7), BBGC2 and BBGC3 were more stable than nisin and CFS of BBGC8. All compounds retained more than 50% of their activity after exposure to 60, 80, 100, and 121°C although the residual activity decreased with increase in the temperature. Therefore, the bacteriocin-like compounds retained their activity despite exposure to different proteolytic, pH and temperature compounds.

### Evidence of Bacteriocin Production in *Clostridium estertheticum* Complex

The CFS of the *C. estertheticum* CF004 was analyzed by UHPLC HRMS and tandem mass spectrometry (MS/MS). Cesin A was detected at 4.41 min with a mass/charge ratio (*m*/*z*) of 709.0051, representing the protonated molecule [M + 3H]^3+^ (Supplementary Figure 8). The calculated molecular formula was C_92_H_145_N_27_O_23_S_4_ (δ of 2.2 ppm), corresponding to the core peptide ITSWSLCTAGCITGRIMGCNK possessing 3 macrocycles, and dehydration at the serine and threonine residues (Supplementary Figure 8). MS/MS analysis was performed and critical fragments were detected, confirming the structure of the natural product (Supplementary Figure 9). The putative sactipeptide estercticin A was detected at 6.54 min with an *m*/*z* of 1148.8243, which represents the charged molecule [M + H + 2Na]^3+^ (Supplementary Figure 10). The calculated molecular formula was C_143_H_226_N_40_O_44_S_6_ (δ of 3.8 ppm) corresponding to the core peptide.

ELNCWACLGCVGCAACVFTLALASAMSALTGSNR bearing three crosslinks. The concentration of the compound was not sufficient to obtain an MS/MS and, therefore, the position of the cycles is unknown.

## Discussion

The shortage of new antimicrobial compounds that are active against multidrug-resistant pathogenic bacteria has reached new heights (Freire-Moran et al., 2011), resulting in a great need for novel antimicrobial compounds that display unique chemistry and mode of action (Miethke et al., 2021). This has drawn attention toward bacteriocins, which are seen as viable alternative to existing antibiotics (Cotter et al., 2013) and increased the attempts to discover novel bacteriocins (Murphy et al., 2011). In the current study, we therefore aimed to uncover the antimicrobial biosynthetic potential of the psychrophilic CEC. Using targeted genome mining for bacteriocin biosynthetic gene clusters (BBGC), we have for the first time revealed widespread distribution of BBGC in CEC after identifying 18 lantibiotic and 2 sactipeptide biosynthetic clusters that were distributed in 18 out of 40 CEC genomes (Table 2). The clusters were further classified into six distinct lantibiotic and 2 sactipeptide clusters based on gene organization highlighting a diverse repertoire of bacteriocins within CEC (Figure 1). At 50%, the frequency of the BBGC was higher than 25% in other anaerobic bacteria, including *Clostridia* spp. (Letzel et al., 2014). Variable frequencies between the BBGC in CEC and other species within *Clostridia* genus suggest CEC is a suitable choice for bacteriocin prospecting within the genus.

Among the well-known members of Class I bacteriocins are the lantibiotics (Class Ia and Ib), and nisin is the most studied lantibiotic (Khelissa et al., 2020). Currently, eleven variants of nisin have been described, including nisin A, F, Q, and Z from *Lactococcus lactis* (Rogers and Whittier, 1928; Mulders et al., 1991; Zendo et al., 2003; De Kwaadsteniet et al., 2008), nisin H, P, U, and U2 from *Streptococcus* spp. (Wirawan et al., 2006; O’Connor et al., 2015; Garcia-Gutierrez et al., 2020), nisin O from *Blautia obeum* (Hatziioanou et al., 2017), nisin J from *Staphylococcus capitis* (O’Sullivan et al., 2020) and Kunkecin A from *Apilactobacillus kunkeei* (Zendo et al., 2020). Through phylogenetic analysis, we discovered a nisin variant, herein identified as cesin A, that is encoded in five *C. estertheticum* genomes (Figure 5). All the currently known nisin variants range from 31 to 39 amino acids (Garcia-Gutierrez et al., 2020). Strikingly, genome and HRMS and MS/MS analyses predicted that cesin A is 21 amino acids and compared to nisin A, it lacks 13 amino acids toward the C-terminus making it the shortest reported natural variant. The C-terminus of nisin is important for antimicrobial activity (Breukink et al., 1997; van Kraaij et al., 1998; Deng et al., 2020), hence further studies are required to elucidate the effect of a short nisin variant on its antimicrobial activity. Other important bacteriocin classes include Class Ib (lantibiotics) and Class Ic (sactipetides) (Alvarez-Sieiro et al., 2016), which were revealed in the current genome mining (Table 2). HRMS analysis also validated the sequence and putative structure of BBGC7-encoded sactipeptide, Estercticin A. The low sequence identity of the CEC bacteriocins confirmed all encoded bacteriocins were novel members of their respective classes and warrant further characterization. The retention of antimicrobial activity of the putative bacteriocins after exposure to different proteolytic, pH and temperature conditions demonstrated their stability making them suitable candidates for application as food preservatives or therapeutic agents.

In most cases, BBGC are encoded in plasmids (Criado et al., 2006). This was also the case for CEC’s BBGC (Supplementary Table 2). Localization of bacteriocins in plasmids enable intraspecies or interspecies horizontal gene transfer (HGT) of the bacteriocins (Lozo et al., 2017), which in turn allows bacteria to acquire advantageous traits relevant for specific niches (Sørensen et al., 2005). The presence of bacteriocins within the plasmids of CEC also suggests a common origin with other Firmicutes as evident with BBGC1, which encodes the variant cesin A. The plasmids carrying BBGC2, which were present in *C. estertheticum*, *C. tagluense*, and genomospecies2, are further evidence of HGT of bacteriocins within CEC and explain the high similarity of the gene clusters. It is particular interesting that some strains including *C. estertheticum* CF004 and CF016 have each acquired two different plasmids encoding different bacteriocins. The relative rarity of these events have been suggested to confer advantages to the producer organism in specific environmental conditions (Main et al., 2020).

## Conclusion

Through targeted genome mining, we identified eight novel bacteriocin biosynthetic gene clusters in strains from the CEC. The clusters belonged to three subclasses of bacteriocins namely, lantibiotics (Class Ia and Ib) and sactipeptides (Class Ic). Complete genome sequencing of 16 CEC strains showed all the gene clusters were plasmid encoded. Following partial purification, phenotypic screening assays linked clusters BBGC1, BBGC2, and BBGC3 with activity against the distantly related gram-positive *B. cereus*, while BBGC2, and BBGC3 were linked with activity against the gram-negative *E. coli*. Furthermore, the partially purified compounds retained activity after exposure to different proteolytic enzymes, temperatures and pH conditions. Evidence of bacteriocin production by CEC was demonstrated through MS/MS and HRMS analysis for the lantibiotic cesin A and sactipeptide estercticin A. The analysis of the data confirmed the key structural feature of cesin A. Estercticin A will be produced in larger amount in order to investigate its structure since it was detected only at low concentration in the CFC. Taken together, the current study reveals that the CEC have evolved to produce stable bacteriocin that can be exploited for application as food preservatives or therapeutic agents. Further studies focused on the optimization, biosynthesis, isolation and characterization of the bacteriocins are proposed.

## Data Availability Statement

The genomes have been deposited in the NCBI under the bioproject PRJNA735695 (https://www.ncbi.nlm.nih.gov/bioproject/PRJNA735695).

## Author Contributions

JW and RS designed the study. MS, NC, and VP carried out whole genome sequencing and assembly. JW carried out the genome mining and phenotypic screening assays and wrote the initial draft manuscript. SS carried out the HRMS and MS/MS analysis. JW, RS, MS, SS, and VP revised the final manuscript. RS supervised the study. All authors contributed to the article and approved the submitted version.

## Conflict of Interest

The authors declare that the research was conducted in the absence of any commercial or financial relationships that could be construed as a potential conflict of interest.

## Publisher’s Note

All claims expressed in this article are solely those of the authors and do not necessarily represent those of their affiliated organizations, or those of the publisher, the editors and the reviewers. Any product that may be evaluated in this article, or claim that may be made by its manufacturer, is not guaranteed or endorsed by the publisher.

## Footnotes

1
http://www.snapgene.com

2
http://www.inkscape.org

## References

[B1] Altschul S. F. Gish W. Miller W. Myers E. W. Lipman D. J. (1990). Basic local alignment search tool. *J. Mol. Biol.* 215 403–410. 10.1016/S0022-2836(05)80360-2 2231712

[B2] Alvarez-Sieiro P. Montalbán-López M. Mu D. Kuipers O. P. (2016). Bacteriocins of lactic acid bacteria: extending the family. *Appl. Microbiol. Biotechnol.* 100 2939–2951. 10.1007/S00253-016-7343-9 26860942 PMC4786598

[B3] Angelopoulou A. Warda A. K. O’Connor P. M. Stockdale S. R. Shkoporov A. N. Field D. (2020). Diverse bacteriocins produced by strains from the human milk microbiota. *Front. Microbiol.* 11:788. 10.3389/FMICB.2020.00788 32508758 PMC7248182

[B4] Arnison P. G. Bibb M. J. Bierbaum G. Bowers A. A. Bugni T. S. Bulaj G. (2013). Ribosomally synthesized and post-translationally modified peptide natural products: overview and recommendations for a universal nomenclature. *Nat. Prod. Rep.* 30 108–160. 10.1039/c2np20085f 23165928 PMC3954855

[B5] Blin K. Shaw S. Kautsar S. A. Medema M. H. Weber T. (2020). The antiSMASH database version 3: increased taxonomic coverage and new query features for modular enzymes. *Nucleic Acids Res.* 49 D639–D643. 10.1093/nar/gkaa978 33152079 PMC7779067

[B6] Brettin T. Davis J. J. Disz T. Edwards R. A. Gerdes S. Olsen G. J. (2015). RASTtk: a modular and extensible implementation of the RAST algorithm for building custom annotation pipelines and annotating batches of genomes. *Sci. Rep.* 5:8365. 10.1038/srep08365 25666585 PMC4322359

[B7] Breukink E. van Kraaij C. Demel R. A. Siezen R. J. Kuipers O. P. de Kruijff B. (1997). The C-terminal region of nisin is responsible for the initial interaction of nisin with the target membrane. *Biochemistry* 36 6968–6976. 10.1021/bi970008u 9188693

[B8] Cebrián R. Macia-Valero A. Jati A. P. Kuipers O. P. (2019). Design and expression of specific hybrid lantibiotics active against pathogenic *Clostridium* spp. *Front. Microbiol.* 10:2154. 10.3389/FMICB.2019.02154 31616392 PMC6768957

[B9] Chen Y. Wang J. Li G. Yang Y. Ding W. (2021). Current advancements in sactipeptide natural products. *Front. Chem.* 9:595991. 10.3389/FCHEM.2021.595991 34095082 PMC8172795

[B10] Collins M. D. (1992). Taxonomic studies on a psychrophilic *Clostridium* from vacuum-packed beef: description of *Clostridium estertheticum* sp. nov. *FEMS Microbiol. Lett.* 96 235–239. 10.1016/0378-1097(92)90410-P 1383083

[B11] Cotter P. D. Hill C. Ross R. P. (2005). Bacteriocins: developing innate immunity for food. *Nat. Rev. Microbiol.* 3 777–788. 10.1038/nrmicro1273 16205711

[B12] Cotter P. D. Ross R. P. Hill C. (2013). Bacteriocins – a viable alternative to antibiotics? *Nat. Rev. Microbiol.* 11 95–105. 10.1038/nrmicro2937 23268227

[B13] Criado R. Diep D. B. Aakra Å Gutiérrez J. Nes I. F. Hernández P. E. (2006). Complete sequence of the enterocin Q-encoding plasmid pCIZ2 from the multiple bacteriocin producer *Enterococcus faecium* L50 and genetic characterization of enterocin Q production and immunity. *Appl. Environ. Microbiol.* 72 6653–6666. 10.1128/AEM.00859-06 17021217 PMC1610292

[B14] Cui Y. Luo L. Wang X. Lu Y. Yi Y. Shan Y. (2021). Mining, heterologous expression, purification, antibactericidal mechanism, and application of bacteriocins: a review. *Compr. Rev. Food Sci. Food Saf.* 20 863–899. 10.1111/1541-4337.12658 33443793

[B15] De Kwaadsteniet M. Ten Doeschate K. Dicks L. M. T. (2008). Characterization of the structural gene encoding nisin F, a new lantibiotic produced by a *Lactococcus lactis* subsp. lactis isolate from freshwater catfish (*Clarias gariepinus*). *Appl. Environ. Microbiol.* 74 547–549. 10.1128/AEM.01862-07 18039827 PMC2223265

[B16] Deegan L. H. Cotter P. D. Hill C. Ross P. (2006). Bacteriocins: biological tools for bio-preservation and shelf-life extension. *Int. Dairy J.* 16 1058–1071. 10.1016/j.idairyj.2005.10.026

[B17] Deng J. Viel J. H. Chen J. Kuipers O. P. (2020). Synthesis and characterization of heterodimers and fluorescent nisin species by incorporation of methionine analogues and subsequent click chemistry. *ACS Synth. Biol.* 9 2525–2536. 10.1021/ACSSYNBIO.0C00308 32786360 PMC7507115

[B18] Dorn-In S. Schwaiger K. Springer C. Barta L. Ulrich S. Gareis M. (2018). Development of a multiplex qPCR for the species identification of *Clostridium estertheticum*, C. *frigoriphilum*, C. *bowmanii* and C. tagluense-like from blown pack spoilage (BPS) meats and from wild boars. *Int. J. Food Microbiol.* 286 162–169. 10.1016/j.ijfoodmicro.2018.08.020 30165293

[B19] Drider D. Fimland G. Héchard Y. McMullen L. M. Prévost H. (2006). The continuing story of class IIa bacteriocins. *Microbiol. Mol. Biol. Rev.* 70 564–582. 10.1128/mmbr.00016-05 16760314 PMC1489543

[B20] Esteves E. Gupta T. B. Whyte P. Brightwell G. Bolton D. (2021). An investigation of the environmental niches of blown pack spoilage causing *Clostridium estertheticum* and *Clostridium gasigenes* on New Zealand beef and sheep farms. *Food Microbiol.* 98:103769. 10.1016/j.fm.2021.103769 33875205

[B21] Esteves E. Whyte P. Gupta T. B. Bolton D. (2020). An investigation of the ecological niches and seasonal nature of *Clostridium estertheticum* and *Clostridium gasigenes* in the Irish beef farm environment. *Lett. Appl. Microbiol.* 71 660–666. 10.1111/lam.13344 32608530

[B22] Flühe L. Marahiel M. A. (2013). Radical S-adenosylmethionine enzyme catalyzed thioether bond formation in sactipeptide biosynthesis. *Curr. Opin. Chem. Biol.* 17 605–612. 10.1016/j.cbpa.2013.06.031 23891473

[B23] Freire-Moran L. Aronsson B. Manz C. Gyssens I. C. So A. D. Monnet D. L. (2011). Critical shortage of new antibiotics in development against multidrug-resistant bacteria-Time to react is now. *Drug Resist. Updat.* 14 118–124. 10.1016/j.drup.2011.02.003 21435939

[B24] Garcia-Gutierrez E. O’Connor P. M. Saalbach G. Walsh C. J. Hegarty J. W. Guinane C. M. (2020). First evidence of production of the lantibiotic nisin P. *Sci. Rep.* 10:3738. 10.1038/s41598-020-60623-0 32111904 PMC7048740

[B25] Hammami R. Zouhir A. Le Lay C. Ben Hamida J. Fliss I. (2010). BACTIBASE second release: a database and tool platform for bacteriocin characterization. *BMC Microbiol.* 10:22. 10.1186/1471-2180-10-22 20105292 PMC2824694

[B26] Hatziioanou D. Gherghisan-Filip C. Saalbach G. Horn N. Wegmann U. Duncan S. H. (2017). Discovery of a novel lantibiotic nisin O from *Blautia obeum* A2-162, isolated from the human gastrointestinal tract. *Microbiology* 163:1292. 10.1099/MIC.0.000515 28857034 PMC5882112

[B27] Khelissa S. Chihib N.-E. Gharsallaoui A. (2020). Conditions of nisin production by *Lactococcus lactis* subsp. lactis and its main uses as a food preservative. *Arch. Microbiol.* 203 465–480. 10.1007/S00203-020-02054-Z 33001222

[B28] Kumariya R. Garsa A. K. Rajput Y. S. Sood S. K. Akhtar N. Patel S. (2019). Bacteriocins: classification, synthesis, mechanism of action and resistance development in food spoilage causing bacteria. *Microb. Pathog.* 128 171–177. 10.1016/j.micpath.2019.01.002 30610901

[B29] Letzel A.-C. Pidot S. J. Hertweck C. (2014). Genome mining for ribosomally synthesized and post-translationally modified peptides (RiPPs) in anaerobic bacteria. *BMC Genomics* 15:983. 10.1186/1471-2164-15-983 25407095 PMC4289311

[B30] Li H. Handsaker B. Wysoker A. Fennell T. Ruan J. Homer N. (2009). The sequence alignment/map format and SAMtools. *Bioinformatics* 25 2078–2079. 10.1093/bioinformatics/btp352 19505943 PMC2723002

[B31] Lozo J. Mirkovic N. O’Connor P. M. Malesevic M. Miljkovic M. Polovic N. (2017). Lactolisterin BU, a novel class II broad-spectrum bacteriocin from *Lactococcus lactis* subsp. lactis bv. diacetylactis BGBU1-4. *Appl. Environ. Microbiol.* 83:e01519-17. 10.1128/AEM.01519-17 28842543 PMC5648901

[B32] Main P. Hata T. Loo T. S. Man P. Novak P. Havlíček V. (2020). Bacteriocin ASM1 is an O/S-diglycosylated, plasmid-encoded homologue of glycocin F. *FEBS Lett.* 594 1196–1206. 10.1002/1873-3468.13708 31829452

[B33] Miethke M. Pieroni M. Weber T. Brönstrup M. Hammann P. Halby L. (2021). Towards the sustainable discovery and development of new antibiotics. *Nat. Rev. Chem.* 5 726–749. 10.1038/s41570-021-00313-1 34426795 PMC8374425

[B34] Mokhtar N. F. K. Hashim A. M. Hanish I. Zulkarnain A. Raja Nhari R. M. H. Abdul Sani A. A. (2020). The Discovery of new antilisterial proteins from *Paenibacillus polymyxa* Kp10 via genome mining and mass spectrometry. *Front. Microbiol.* 11:960. 10.3389/FMICB.2020.00960 32714281 PMC7343975

[B35] Mokoena M. P. (2017). Lactic acid bacteria and their bacteriocins: classification, biosynthesis and applications against uropathogens: a mini-review. *Molecules* 22:1255. 10.3390/molecules22081255 28933759 PMC6152299

[B36] Mulders J. W. M. Boerrigter I. J. Rollema H. S. Siezen R. J. de Vos W. M. (1991). Identification and characterization of the lantibiotic nisin Z, a natural nisin variant. *Eur. J. Biochem.* 201 581–584. 10.1111/J.1432-1033.1991.TB16317.X 1935953

[B37] Murphy K. O’Sullivan O. Rea M. C. Cotter P. D. Ross R. P. Hill C. (2011). Genome mining for radical SAM protein determinants reveals multiple sactibiotic-like gene clusters. *PLoS One* 6:e20852. 10.1371/JOURNAL.PONE.0020852 21760885 PMC3132745

[B38] O’Connor P. M. O’Shea E. F. Guinane C. M. O’Sullivan O. Cotter P. D. Ross R. P. (2015). Nisin H is a new nisin variant produced by the gut-derived strain *Streptococcus hyointestinalis* DPC6484. *Appl. Environ. Microbiol.* 81 3953–3960. 10.1128/AEM.00212-15 25841003 PMC4524162

[B39] O’Sullivan J. N. O’Connor P. M. Rea M. C. O’Sullivan O. Walsh C. J. Healy B. (2020). Nisin J, a novel natural nisin variant, is produced by *Staphylococcus capitis* sourced from the human skin microbiota. *J. Bacteriol.* 202:e00639-19. 10.1128/JB.00639-19 31740495 PMC6964739

[B40] Pahalagedara A. S. N. W. Flint S. Palmer J. Brightwell G. Gupta T. B. (2020). Antimicrobial production by strictly anaerobic *Clostridium* spp. *Int. J. Antimicrob. Agents* 55:105910. 10.1016/j.ijantimicag.2020.105910 31991218

[B41] Palevich N. Palevich F. P. Maclean P. H. Altermann E. Gardner A. Burgess S. (2021). Comparative genomics of *Clostridium* species associated with vacuum-packed meat spoilage. *Food Microbiol.* 95:103687. 10.1016/j.fm.2020.103687 33397617

[B42] Rogers L. A. Whittier E. O. (1928). Limiting factors in the lactic fermentation. *J. Bacteriol.* 16 211–229. 10.1128/jb.16.4.211-229.1928 16559334 PMC375023

[B43] Shcherbakova V. A. Chuvilskaya N. A. Rivkina E. M. Pecheritsyna S. A. Laurinavichius K. S. Suzina N. E. (2005). Novel psychrophilic anaerobic spore-forming bacterium from the overcooled water brine in permafrost: description *Clostridium algoriphilum* sp. nov. *Extremophiles* 9 239–246. 10.1007/s00792-005-0438-3 15844014

[B44] Sørensen S. J. Bailey M. Hansen L. H. Kroer N. Wuertz S. (2005). Studying plasmid horizontal transfer in situ: a critical review. *Nat. Rev. Microbiol.* 3 700–710. 10.1038/nrmicro1232 16138098

[B45] Spring S. (2003). Characterization of novel psychrophilic clostridia from an Antarctic microbial mat: description of *Clostridium frigoris* sp. nov., *Clostridium lacusfryxellense* sp. nov., *Clostridium bowmanii* sp. nov. and *Clostridium psychrophilum* sp. nov. and reclassification of *Clostridium laramiense* as *Clostridium estertheticum* subsp. laramiense subsp. nov. *Int. J. Syst. Evol. Microbiol.* 53 1019–1029. 10.1099/ijs.0.02554-0 12892121

[B46] Suetin S. V. Shcherbakova V. A. Chuvilskaya N. A. Rivkina E. M. Suzina N. E. Lysenko A. M. (2009). *Clostridium tagluense* sp. nov., a psychrotolerant, anaerobic, spore-forming bacterium from permafrost. *Int. J. Syst. Evol. Microbiol.* 59 1421–1426. 10.1099/ijs.0.002295-0 19502327

[B47] Tracanna V. de Jong A. Medema M. H. Kuipers O. P. (2017). Mining prokaryotes for antimicrobial compounds: from diversity to function. *FEMS Microbiol. Rev.* 41 417–429. 10.1093/femsre/fux014 28402441

[B48] Van Heel A. J. De Jong A. Song C. Viel J. H. Kok J. Kuipers O. P. (2018). BAGEL4: a user-friendly web server to thoroughly mine RiPPs and bacteriocins. *Nucleic Acids Res.* 46 W278–W281. 10.1093/nar/gky383 29788290 PMC6030817

[B49] van Kraaij C. Breukink E. Noordermeer M. A. Demel R. A. Siezen R. J. Kuipers O. P. (1998). Pore formation by nisin involves translocation of its c-terminal part across the membrane. *Biochemistry* 37 16033–16040. 10.1021/bi980931b 9819196

[B50] Vezina B. Rehm B. H. A. Smith A. T. (2020). Bioinformatic prospecting and phylogenetic analysis reveals 94 undescribed circular bacteriocins and key motifs. *BMC Microbiol.* 20:77. 10.1186/S12866-020-01772-0 32252629 PMC7132975

[B51] Wambui J. Stephan R. (2019). Relevant aspects of *Clostridium estertheticum* as a specific spoilage organism of vacuum-packed meat. *Microorganisms* 7:142. 10.3390/microorganisms7050142 31137543 PMC6560419

[B52] Wambui J. Cernela N. Corti S. Stephan R. (2020a). Comparative genome analysis and phenotypic characterization of *Clostridium gasigenes* CGAS001 isolated from chilled vacuum-packed lamb meat. *Front. Microbiol.* 11:2048. 10.3389/FMICB.2020.02048 32983035 PMC7476324

[B53] Wambui J. Cernela N. Stevens M. J. A. Ghielmetti G. Stephan R. (2020b). Draft genome sequences of two *Clostridium algidicarnis* strains isolated from meat juice samples of chilled vacuum-packed lamb meat. *Microbiol. Resour. Announc.* 9 19–20. 10.1128/mra.00983-20 33154005 PMC7645660

[B54] Wambui J. Cernela N. Stevens M. J. A. Stephan R. (2020c). Draft genome sequence of *Clostridium estertheticum* CEST001, belonging to a novel subspecies of C. *estertheticum*, isolated from chilled vacuum-packed lamb meat imported to Switzerland. *Microbiol. Resour. Announc.* 9 13–14. 10.1128/MRA.00806-20 32817160 PMC7427198

[B55] Wambui J. Püntener S. Corti S. Cernela N. Stephan R. (2020d). Detection of psychrophilic *Clostridium* spp. causing “blown pack” spoilage in meat juice samples from chilled vacuum-packed beef and lamb meat imported from different countries to Switzerland. *J. Food Prot.* 83 56–59. 10.4315/0362-028X.JFP-19-321 31825674

[B56] Wambui J. Cernela N. Stevens M. J. A. Stephan R. (2021a). Whole genome sequence-based identification of *Clostridium estertheticum* complex strains supports the need for taxonomic reclassification within the species *Clostridium estertheticum*. *Front. Microbiol.* 12:727022. 10.3389/fmicb.2021.727022 34589074 PMC8473909

[B57] Wambui J. Ghielmetti G. Morach M. Hochreutener M. Stephan R. (2021b). Detection of psychrophilic *Clostridium* spp. in fecal samples from cattle of different ages sampled at the slaughterhouse level. *J. Food Prot.* 84 58–62. 10.4315/JFP-20-259 32818242

[B58] Wick R. R. Judd L. M. Gorrie C. L. Holt K. E. (2017). Unicycler: resolving bacterial genome assemblies from short and long sequencing reads. *PLoS Comput. Biol.* 13:e1005595. 10.1371/journal.pcbi.1005595 28594827 PMC5481147

[B59] Williams A. N. Sorout N. Cameron A. J. Stavrinides J. (2020). The integration of genome mining, comparative genomics, and functional genetics for biosynthetic gene cluster identification. *Front. Genet.* 11:1543. 10.3389/FGENE.2020.600116 33343637 PMC7744662

[B60] Wirawan R. E. Klesse N. A. Jack R. W. Tagg J. R. (2006). Molecular and genetic characterization of a novel nisin variant produced by *Streptococcus uberis*. *Appl. Environ. Microbiol.* 72 1148–1156. 10.1128/AEM.72.2.1148-1156.2006 16461661 PMC1392965

[B61] Zendo T. Fukao M. Ueda K. Higuchi T. Nakayama J. Sonomoto K. (2003). Identification of the lantibiotic nisin Q, a new natural nisin variant produced by *Lactococcus lactis* 61-14 isolated from a river in japan. *Biosci. Biotechnol. Biochem.* 67 1616–1619. 10.1271/BBB.67.1616 12913315

[B62] Zendo T. Ohashi C. Maeno S. Piao X. Salminen S. Sonomoto K. (2020). Kunkecin A, a new nisin variant bacteriocin produced by the fructophilic lactic acid bacterium, *Apilactobacillus kunkeei* FF30-6 isolated from honey bees. *Front. Microbiol.* 11:2130. 10.3389/fmicb.2020.571903 33042078 PMC7525160

[B63] Zhong Z. He B. Li J. Li Y. X. (2020). Challenges and advances in genome mining of ribosomally synthesized and post-translationally modified peptides (RiPPs). *Synth. Syst. Biotechnol.* 5 155–172. 10.1016/J.SYNBIO.2020.06.002 32637669 PMC7327761

