# Supplementary material for: Targeted Genome Mining Reveals the Psychrophilic Clostridium estertheticum Complex as a Potential Source for Novel Bacteriocins, Including Cesin A and Estercticin A
Source: Front Microbiol. 2022 Jan 13;12:801467. doi: 10.3389/fmicb.2021.801467 (PMC8792950; doi:10.3389/fmicb.2021.801467)
Supplement: Supplementary file 1 [file Table_1.docx]

Supplementary Material

**Supplementary Table 1**: List of *C. estertheticum* complex genomes. Genomes in bold were excluded from the study due to high genetic relatedness with other genomes

| Species | Strain ID | Source | Origin and/or isolation | Genbank ID |
| --- | --- | --- | --- | --- |
| *C. bowmanii* | DSM 14206 | Microbial mat | Antarctica | GCA_018861315.1 |
| *C. estertheticum* | CEST001 | Lamb | New Zealand | GCA_013093435.1 |
| *C. estertheticum* | CF001 | Bovine feces | Switzerland | GCA_019537155.1 |
| *C. estertheticum* | CF002 | Bovine feces | Switzerland | GCA_018861295.1 |
| *C. estertheticum* | CF003 | Bovine feces | Switzerland | GCA_018861435.1 |
| *C. estertheticum* | CF004 | Bovine feces | Switzerland | GCA_019661245.1 |
| *C. estertheticum* | CF005 | Bovine feces | Switzerland | GCA_019661205.1 |
| *C. estertheticum* | CF006 | Bovine feces | Switzerland | GCA_018861375.1 |
| *C. estertheticum* | CF007 | Bovine feces | Switzerland | GCA_019661225.1 |
| *C. estertheticum* | CF008 | Bovine feces | Switzerland | GCA_019537135.1 |
| *C. estertheticum* | CF009 | Bovine feces | Switzerland | GCA_018861325.1 |
| *C. estertheticum* | CF010 | Bovine feces | Switzerland | GCA_018861855.1 |
| *C. estertheticum* | CF013 | Bovine feces | Switzerland | GCA_018861795.1 |
| *C. estertheticum* | CF015 | Bovine feces | Switzerland | GCA_020443505.1 |
| *C. estertheticum* | CF016 | Bovine feces | Switzerland | GCA_020443575.1 |
| *C. estertheticum* | CM018 | Lamb | New Zealand | GCA_018861665.1 |
| ***C. estertheticum*** | **CM020** | **Lamb** | **New Zealand** | **GCA_018861305.1** |
| ***C. estertheticum*** | **CM032** | **Beef** | **Switzerland** | **GCA_020443525.1** |
| ***C. estertheticum*** | **CM033** | **Horse meat** | **Spain** | **GCA_020443465.1** |
| *C. estertheticum* | CM034 | Horse meat | Spain | GCA_020443485.1 |
| *C. estertheticum* | CM035 | Horse meat | Spain | GCA_020443435.1 |
| *C. estertheticum* | DSM 14864 | Beef | USA | GCA_008933175.1 |
| *C. estertheticum* | DSM 8809 | Meat | UK | GCA_001877035.1 |
| ***C. estertheticum*** | **MA19** | **Pork** | **Canada** | **GCA_009295575.1** |
| *C. estertheticum* | MA41 | Pork | Canada | GCA_009295545.1 |
| *C. estertheticum* | DSM 17811 | Microbial mat | Antarctica | GCA_018861905.1 |
| *C. algoriphilum* | DSM 16153 | Permafrost | Russia | GCA_020443705.1 |
| *C. frigoris* | DSM 14204 | Microbial mat | Antarctica | GCA_018861865.1 |
| *C. lacusfryxellense* | DSM 14205 | Microbial mat | Antarctica | GCA_018861735.1 |
| *C. psychrophilum* | DSM 14207 | Permafrost | Russia | GCA_018861705.1 |
| *C. tagluense* | CM008 | Lamb | New Zealand | GCA_019537185.1 |
| ***C. tagluense*** | **CM019** | **Lamb** | **New Zealand** | **GCA_020443605.1** |
| ***C. tagluense*** | **CM021** | **Lamb** | **Ireland** | **GCA_020443795.1** |
| *C. tagluense* | CM022 | Lamb | Ireland | GCA_020443665.1 |
| ***C. tagluense*** | **CM023** | **Beef** | **Lithuania** | **GCA_020443545.1** |
| *C. tagluense* | CM024 | Beef | Switzerland | GCA_018861655.1 |
| ***C. tagluense*** | **CM025** | **Lamb** | **New Zealand** | **GCA_020443745.1** |
| ***C. tagluense*** | **CM031** | **Lamb** | **New Zealand** | **GCA_020443725.1** |
| ***C. tagluense*** | **CF014** | **Bovine feces** | **Switzerland** | **GCA_020443645.1** |
| *C. tagluense* | CS002 | Equipment | Switzerland | GCA_020443625.1 |
| *C. tagluense* | DSM 17763 | Permafrost | Canada | GCA_003865095.1 |
| *C. tagluense* | FP1 | Lamb | New Zealand | GCA_011065975.1 |
| *C. tagluense* | FP2 | Venison | New Zealand | GCA_011065955.1 |
| *Clostridium* spp. | CM027 | Lamb | Ireland | GCA_019537145.1 |
| *Clostridium* spp. | CM028 | Lamb | Ireland | GCA_019537175.1 |
| *Clostridium* spp. | CF011 | Bovine feces | Switzerland | GCA_018861595.1 |
| *Clostridium* spp. | CF012 | Bovine feces | Switzerland | GCA_018861755.1 |
| *Clostridium* spp. | CS001 | Equipment | Switzerland | GCA_020443565.1 |
| *Clostridium* spp. | FP3 | Lamb | New Zealand | GCA_011065935.1 |
| *Clostridium* spp. | FP4 | Lamb | New Zealand | GCA_011065905.1 |

**Supplementary Table 2**: List of 16 complete genomes sequenced in the current study for purposes of fully mapping *C. estertheticum* complex bacteriocin biosynthetic gene clusters

| Species | Strain ID |
| --- | --- |
| *C. estertheticum* | CEST001 |
| *C. estertheticum* | CF001 |
| *C. estertheticum* | CF003 |
| *C. estertheticum* | CF004 |
| *C. estertheticum* | CF005 |
| *C. estertheticum* | CF007 |
| *C. estertheticum* | CF008 |
| *C. estertheticum* | CF009 |
| *C. estertheticum* | CF016 |
| *C. estertheticum* | CM034 |
| *C. estertheticum* | DSM 14864 |
| *C. tagluense* | CM008 |
| *C. tagluense* | CM022 |
| *Clostridium* spp. | CF011 |
| *Clostridium* spp. | CM027 |
| *Clostridium* spp. | CM028 |

**Supplementary Table 3**: Antimicrobial activity profile of cell free supernatants of *C. estertheticum* complex strains possessing bacteriocin biosynthetic gene clusters BBGC1. All strains belonged to species *C. estertheticum*

| Strain ID | Species | CF004* | CF001 | CEST001 | CF007 | CF008 | CM034 |
| --- | --- | --- | --- | --- | --- | --- | --- |
| CF010 | *C. estertheticum* | + | - | - | - | - | - |
| CM024 | *C. tagluense* | ++ | - | - | - | - | - |
| CF012 | Genomospecies3 | + | + | + | + | - | - |
| DSM 14205 | *C. lacusfryxellense* | ++++ | +++ | +++ | +++ | - | - |
| DSM 14206 | *C. bowmanii* | ++++ | ++ | ++ | ++ | - | - |
| DSM 14204 | *C. frigoris* | ++++ | ++++ | ++++ | ++++ | - | - |
| CM003 | *C. algidicarnis* | ++ | - | - | - | - | - |
| CM005 | *C. gasigenes* | ++ | ++ | + | ++ | - | - |
| CH_85 | *B. cereus* | - | - | - | - | - | - |
| MNZ1 | *S. aureus* | - | - | - | - | - | - |
| EGDe | *L. monocytogenes* | - | - | - | - | - | - |
| GH24 | *E. faecalis* | - | - | - | - | - | - |
| ATCC 25922 | *E. coli* | - | - | - | - | - | - |
| ATCC 27853 | *P. aeruginosa* | - | - | - | - | - | - |
| NZ 6242-90 | *E. cloacae* | - | - | - | - | - | - |
| NZ 513-92 | *K. pneumoniae* | - | - | - | - | - | - |

Inhibition zone (mm): - = No inhibition zone; + = 12-20; ++ = 21-30; +++ = 31-40; ++++ => 40

^*^Also harbors BBGC7

**Supplementary Table 4:** Antimicrobial activity profile of cell free supernatants of *C. estertheticum* complex strains possessing bacteriocin biosynthetic gene clusters BBGC2*

| Strain ID | Species | CM008† | DSM 14864^§^ | CM027^#^ | CM028^#^ | CF011^#^ |
| --- | --- | --- | --- | --- | --- | --- |
| CF010 | *C. estertheticum* | - | - | + | + | + |
| CM024 | *C. tagluense* | - | - | - | - | - |
| CF012 | Genomospecies3 | - | - | - | - | - |
| DSM 14205 | *C. lacusfryxellense* | - | - | +++ | +++ | +++ |
| DSM 14206 | *C. bowmanii* | - | - | ++++ | ++++ | +++ |
| DSM 14204 | *C. frigoris* | - | - | ++++ | ++++ | ++++ |
| CM003 | *C. algidicarnis* | - | - | + | + | + |
| CM005 | *C. gasigenes* | - | - | - | - | - |
| CH_85 | *B. cereus* | - | - | - | - | - |
| MNZ1 | *S. aureus* | - | - | - | - | - |
| EGDe | *L. monocytogenes* | - | - | - | - | - |
| GH24 | *E. faecalis* | - | - | - | - | - |
| ATCC 25922 | *E. coli* | - | - | - | - | - |
| ATCC 27853 | *P. aeruginosa* | - | - | - | - | - |
| NZ 6242-90 | *E. cloacae* | - | - | - | - | - |
| NZ 513-92 | *K. pneumoniae* | - | - | - | - | - |

Inhibition zone (mm): - = No inhibition zone; + = 12-20; ++ = 21-30; +++ = 31-40; ++++ => 40

**C.* *estertheticum* CF016 failed to grow while *C.* *estertheticum* MA19 was not available in-house

†*C. tagluense*

^§^*C. estertheticum*

^#^Genomospecies2

**Supplementary Table 5:** Antimicrobial activity profile of cell free supernatants of *C. estertheticum* complex strains possessing bacteriocin biosynthetic gene clusters BBGC3, BBGC4 and BBGC8

|  |  | BBGC3 | | BBGC4 | BBGC8 |
| --- | --- | --- | --- | --- | --- |
| Strain ID | Species | CF003^§^ | CF009^§^ | CF005^§^ | CM022^§^† |
| CF010 | *C. estertheticum* | - | - | - | - |
| CM024 | *C. tagluense* | - | - | - | - |
| CF012 | Genomospecies3 | - | - | - | - |
| DSM 14205 | *C. lacusfryxellense* | - | - | - | ++ |
| DSM 14206 | *C. bowmanii* | ++ | ++ | - | ++ |
| DSM 14204 | *C. frigoris* | ++++ | ++++ | - | ++ |
| CM003 | *C. algidicarnis* | - | - | - | - |
| CM005 | *C. gasigenes* | - | - | - | - |
| CH_85 | *B. cereus* | - | - | - | - |
| MNZ1 | *S. aureus* | - | - | - | - |
| EGDe | *L. monocytogenes* | - | - | - | - |
| GH24 | *E. faecalis* | - | - | - | - |
| ATCC 25922 | *E. coli* | - | - | - | - |
| ATCC 27853 | *P. aeruginosa* | - | - | - | - |
| NZ 6242-90 | *E. cloacae* | - | - | - | - |
| NZ 513-92 | *K. pneumoniae* | - | - | - | - |

Inhibition zone (mm): - = No inhibition zone; + = 12-20; ++ = 21-30; +++ = 31-40; ++++ => 40

^§^*C. estertheticum*

†*C. tagluense*


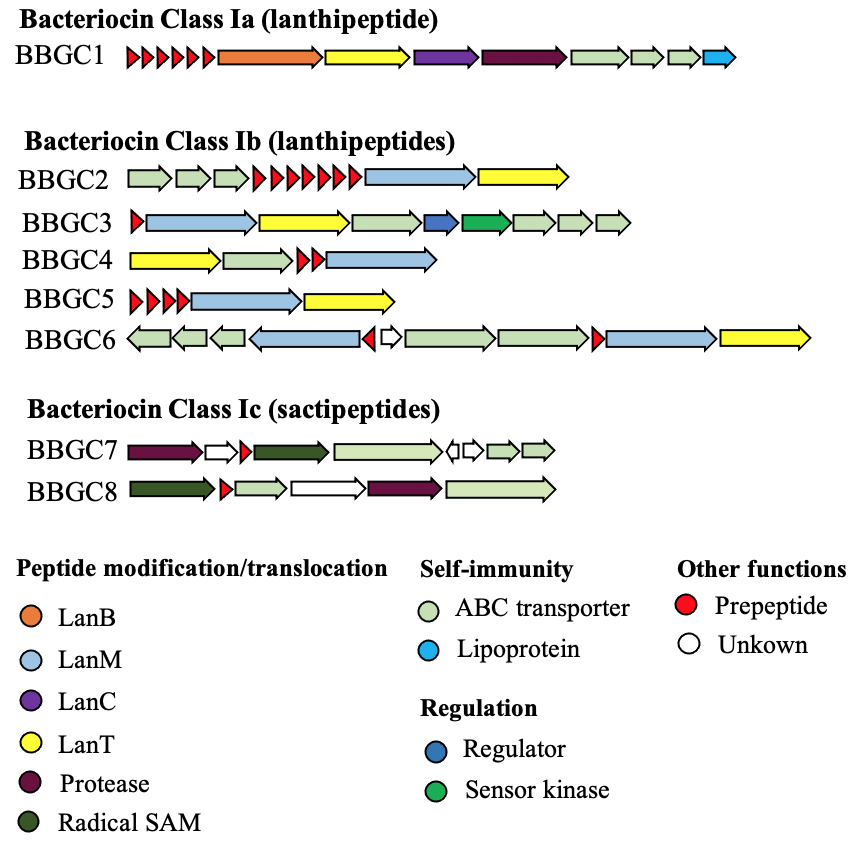

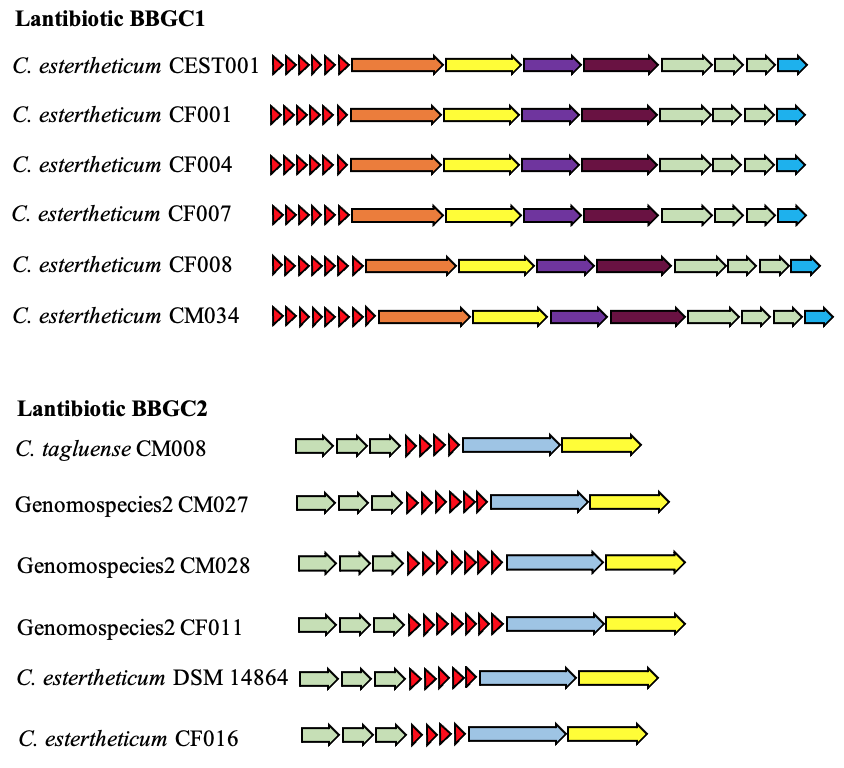


**Supplementary Figure 1**: Diversity of bacteriocin biosynthetic gene clusters BBGC1 (lantibiotic Class Ia) and BBGC2 (lantibiotic Class Ib) identified within *C. estertheticum* complex. The gene clusters were not drawn to scale.

**Supplementary Figure 2**: Localization of Class Ia bacteriocin biosynthetic gene clusters in the plasmids of *C. estertheticum* strains (A) CF001, (B) CEST001, (C) CF004, (D) CF008 and (E) CF007


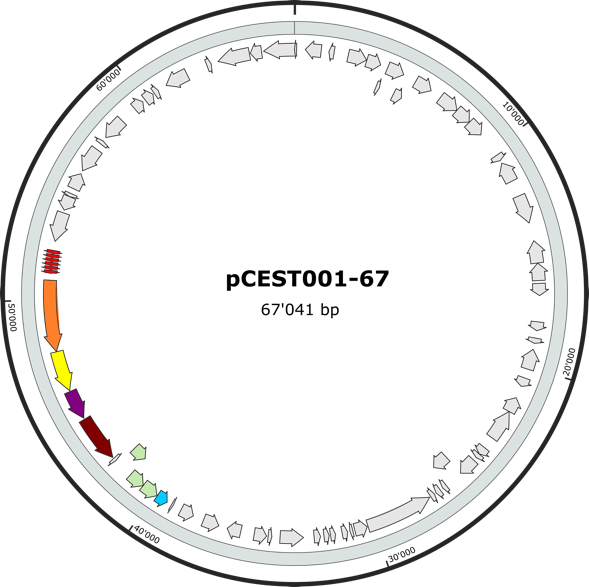

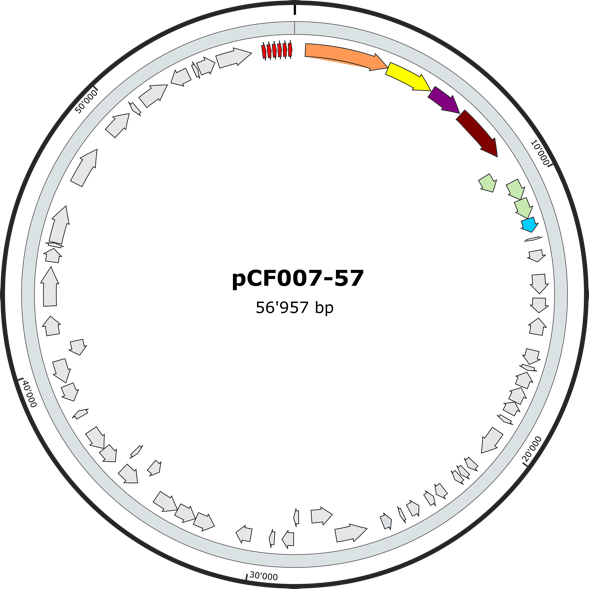

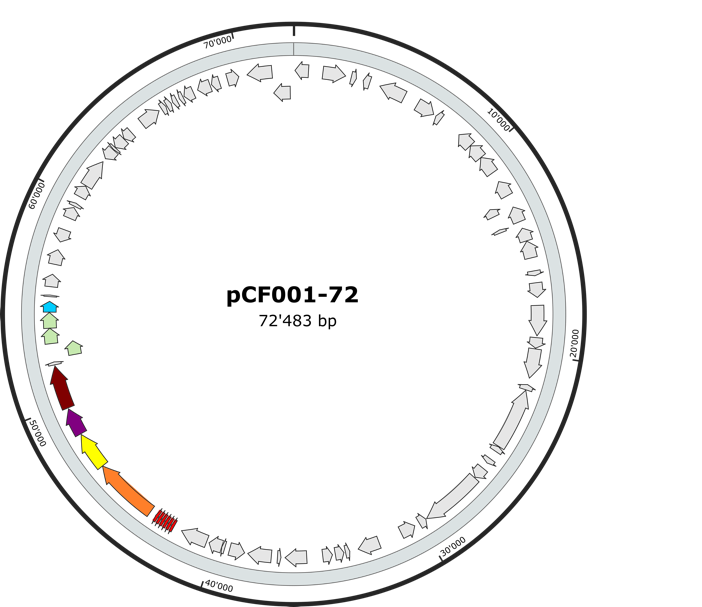

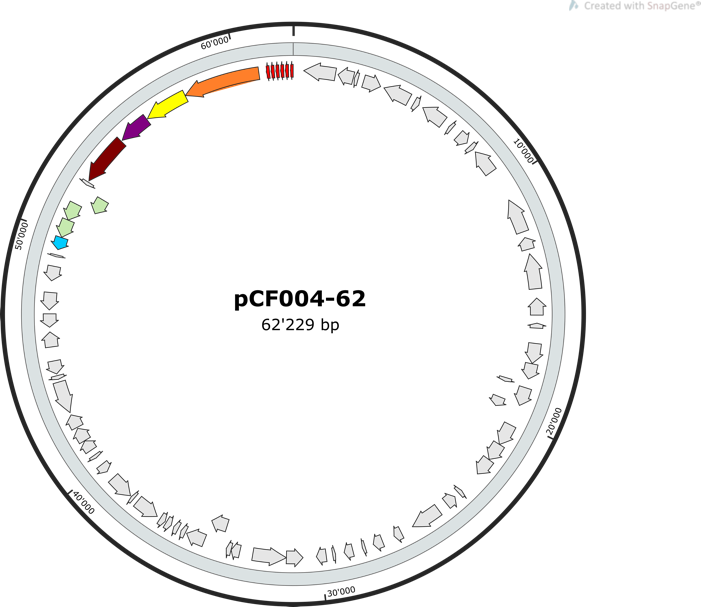

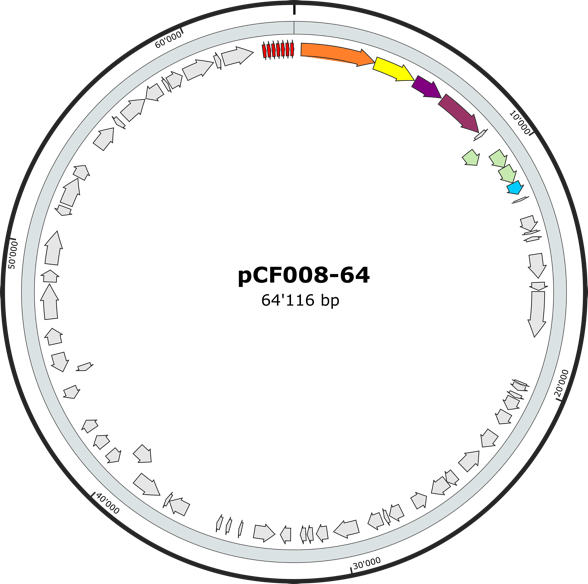


C

D

A

B

E

**Supplementary Figure 3**: Localization of Class Ib bacteriocin biosynthetic gene clusters in the plasmids of (A) *C. tagluense* CM008, (B) *C. estertheticum* DSM 14864 and Genomospecies2 strains (C) CM027, (D) CM028 and (E) CF011


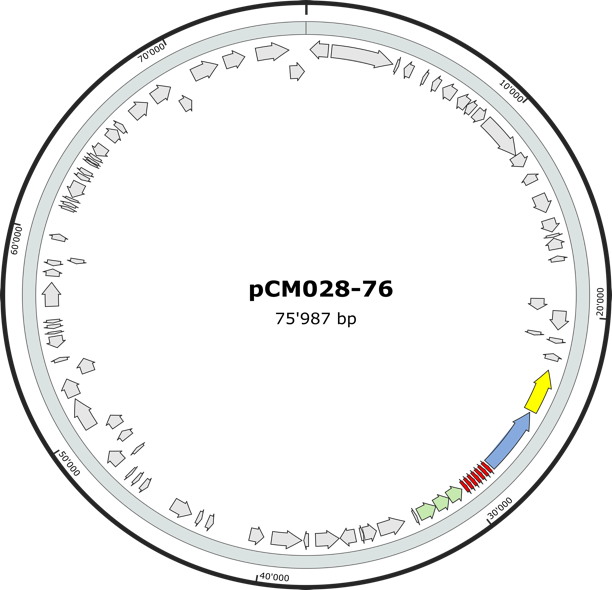

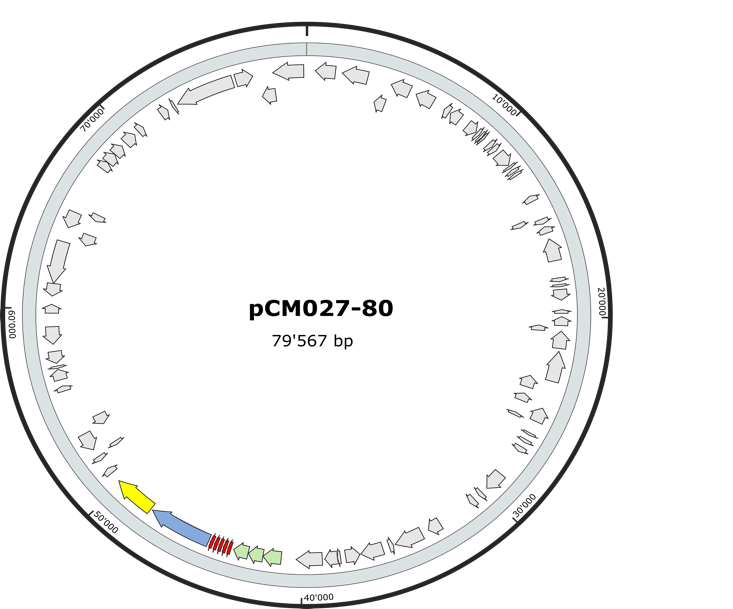

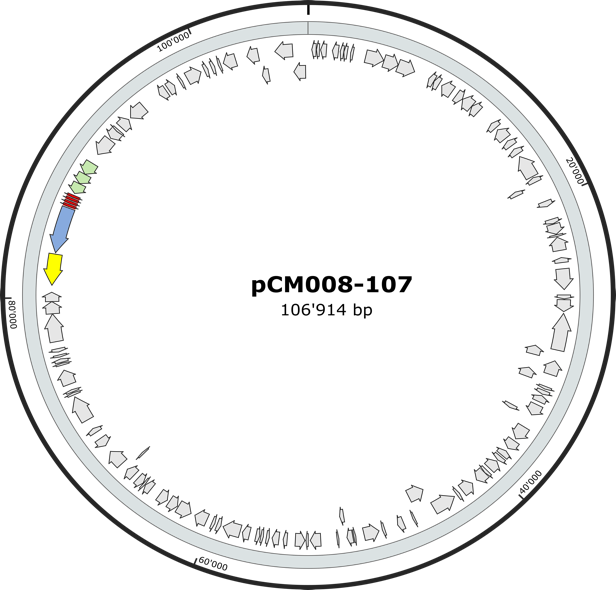

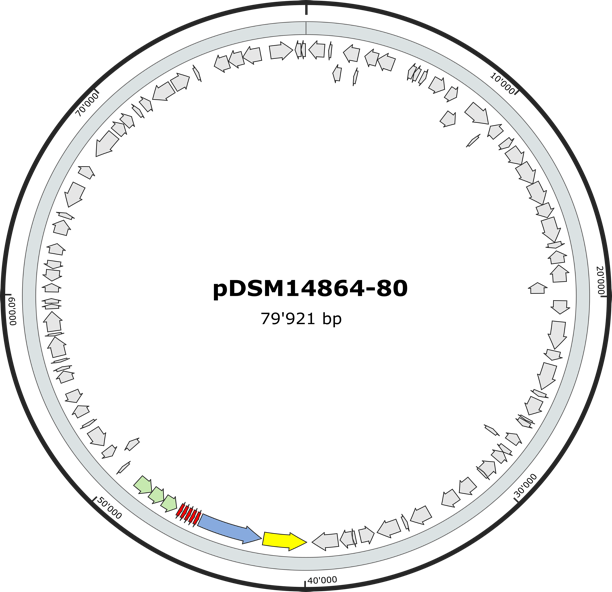

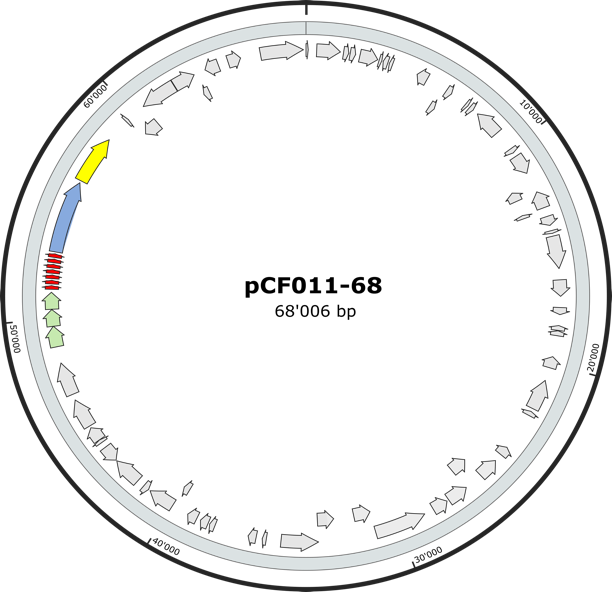


C

D

A

B

E


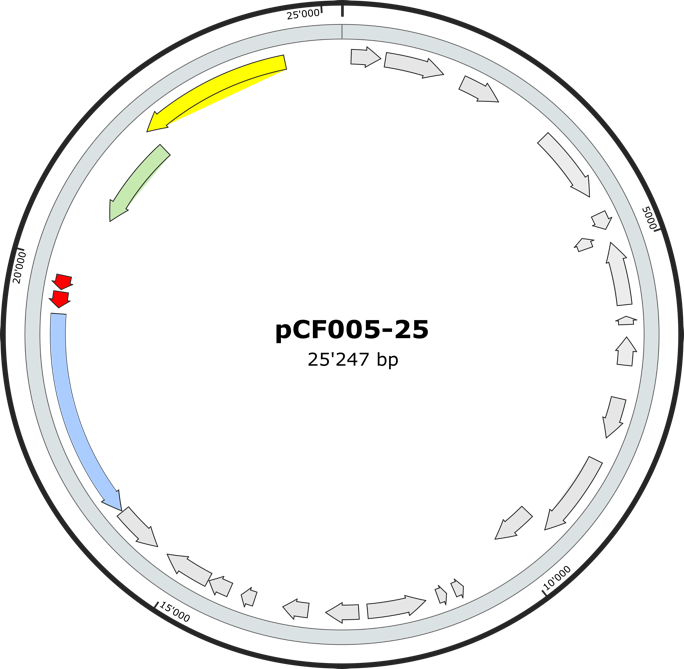


A

B


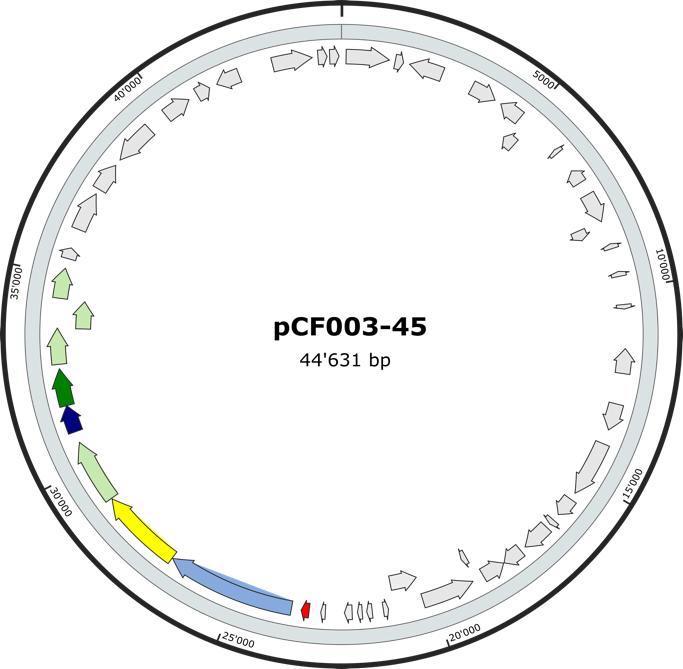


**Supplementary Figure 4**: Localization of Class Ib bacteriocin biosynthetic gene clusters in the plasmids *C. estertheticum* strains (A) CF003 and (B) CF005


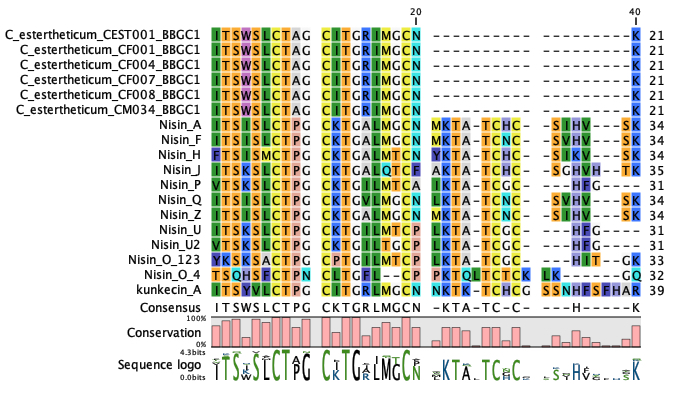


**Supplementary Figure 5:** Sequence alignment of the corepeptides of cesin A (*C. estertheticum* BBGC1) from five strains and 10 known natural variants of nisin. With 21 amino acids, cesin A is the shortest natural variant of nisin. It is characterized by five unique amino acids (shown with blue arrows) in the N-terminus and a short C-terminus.

**
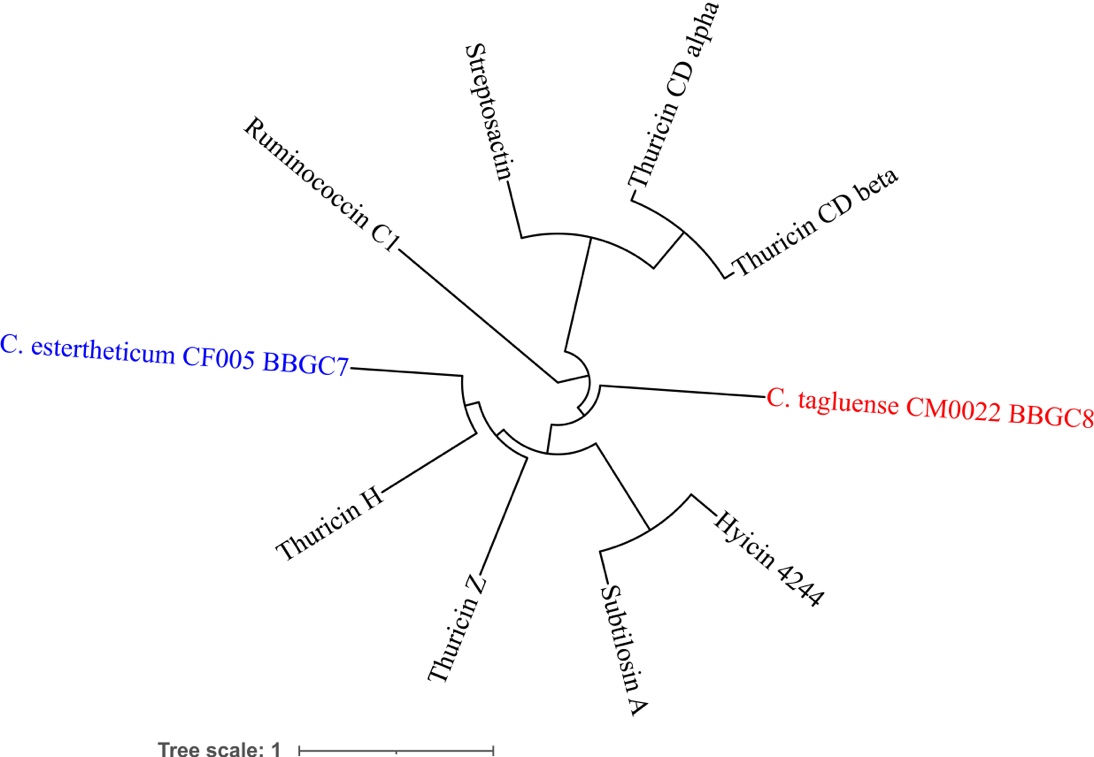
**

**Supplementary Figure 6**. Phylogenetic relatedness of *Clostridium estertheticum* complex sactipeptides and known sactipeptides. The BBGC7 encoded sactipeptide (blue font) clustered with thuricin H while the BBGC8 sactipeptides (red font) did not cluster with any known sactipeptides. The bar indicates 1 substitution per nucleotide position.


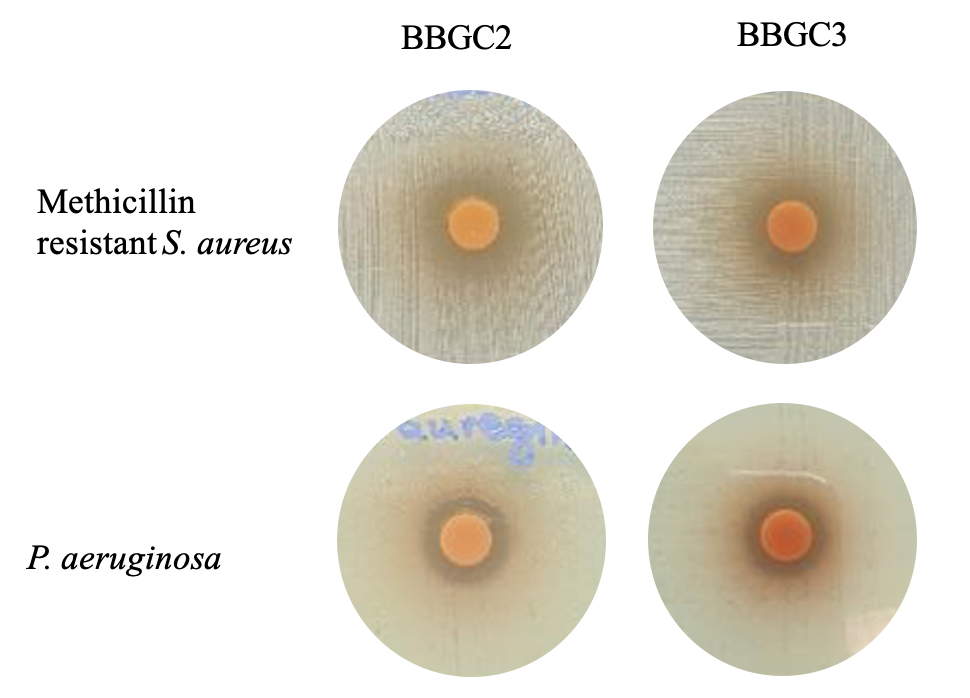


**Supplementary Figure 7.** Antimicrobial activity of partially purified extracts of *C. tagluense* CM008 (BBGC2) and *C. estertheticum* CF009 (BBGC3), respectively, against methicillin resistant *S. aureus* and *P. aeruginosa*.


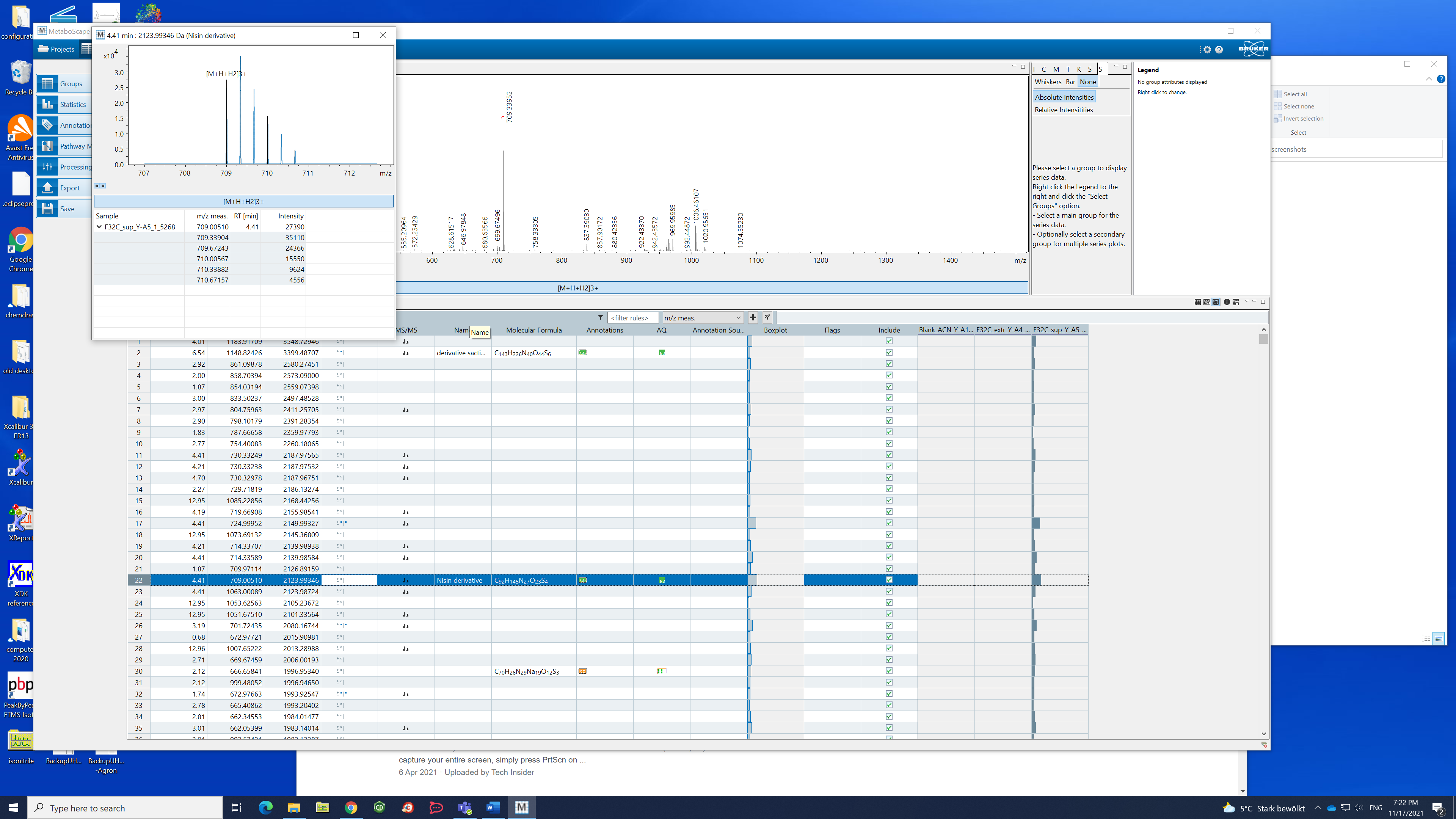


IleDhbAlaTrpDhaLeuAlaAbuAlaGlyAlaIleAbuGlyArgIleMetGlyAlaAsnLys

S

S

S

**Supplementary Figure 8.** HRMS and predicted structure of cesin A detected in the supernatant of *C. estertheticum* CF004. Abbreviation: Dhb (dehydrobutyrine), Dha (dehydroalanine) and Abu (aminobutyric acid):


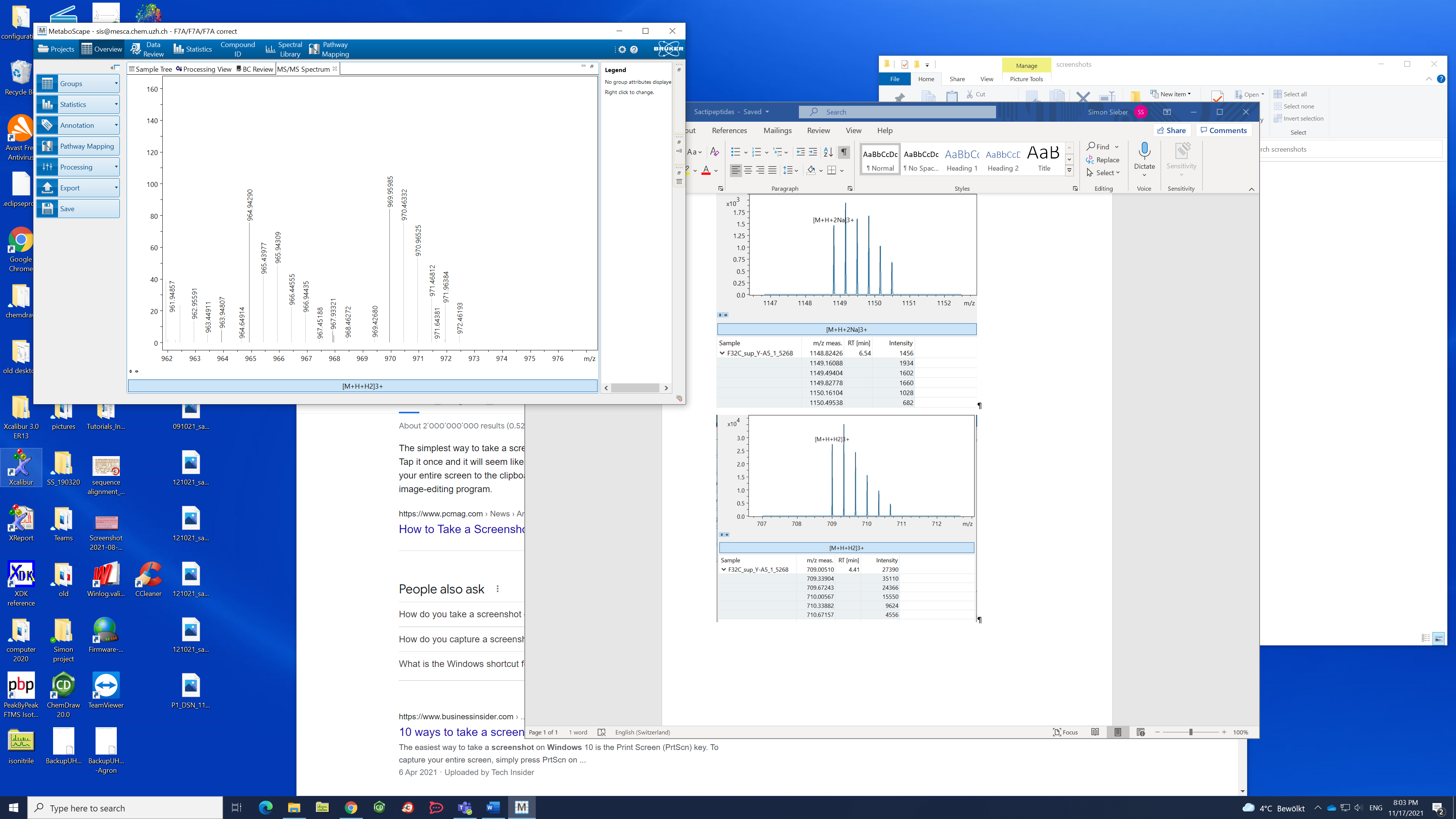


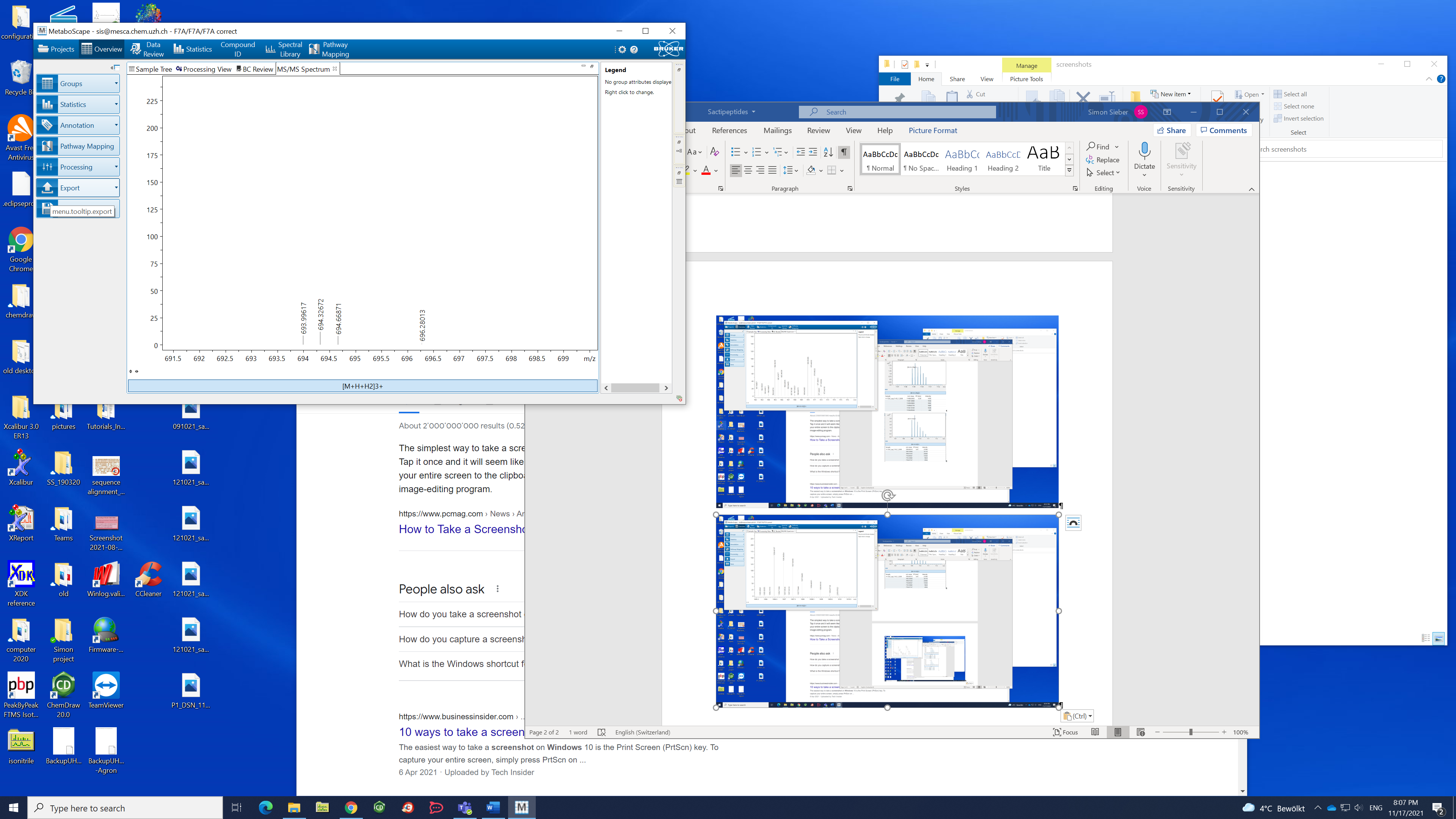


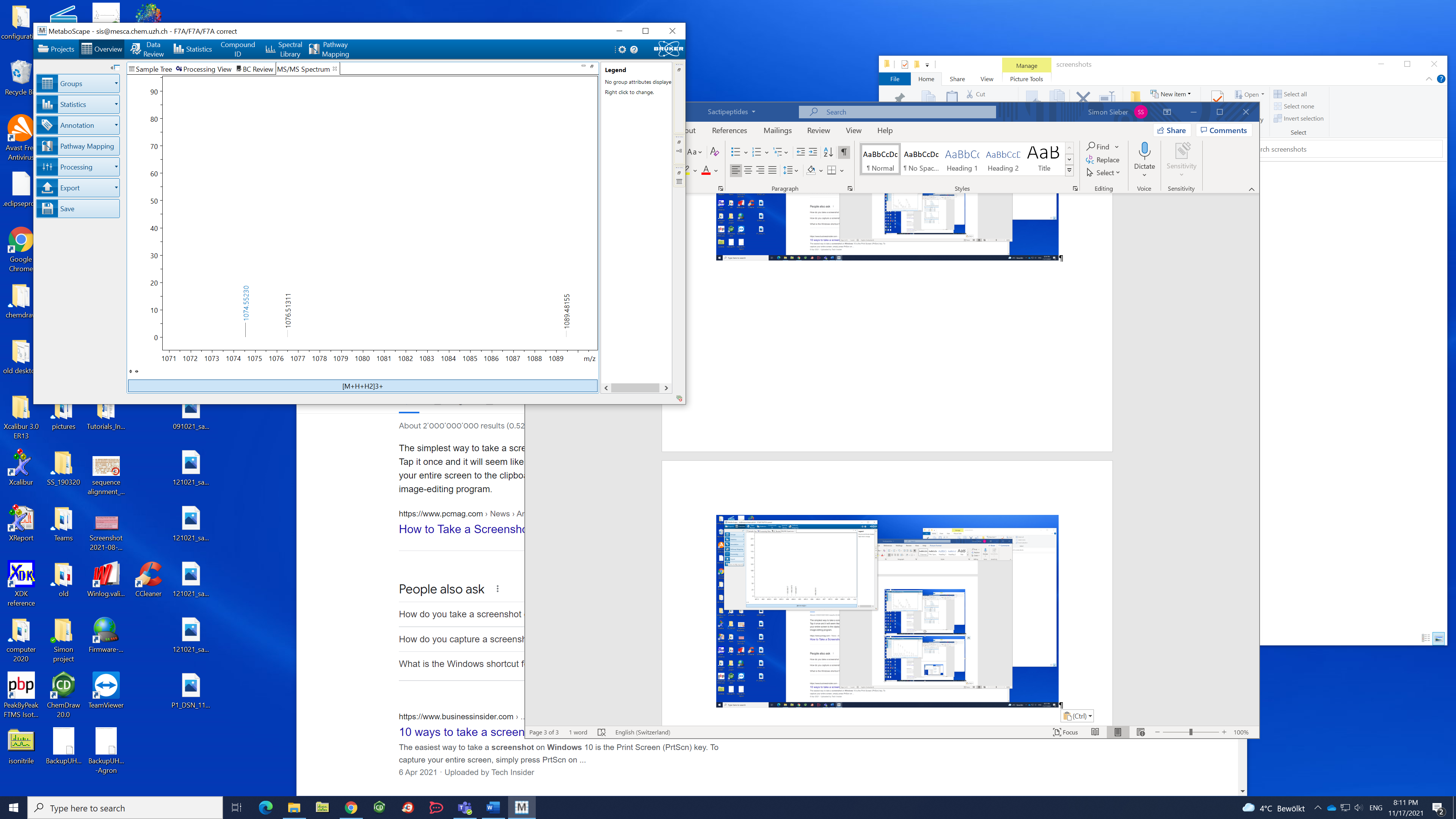


**Supplementary Figure 9.** MS/MS of cesin A key fragments.


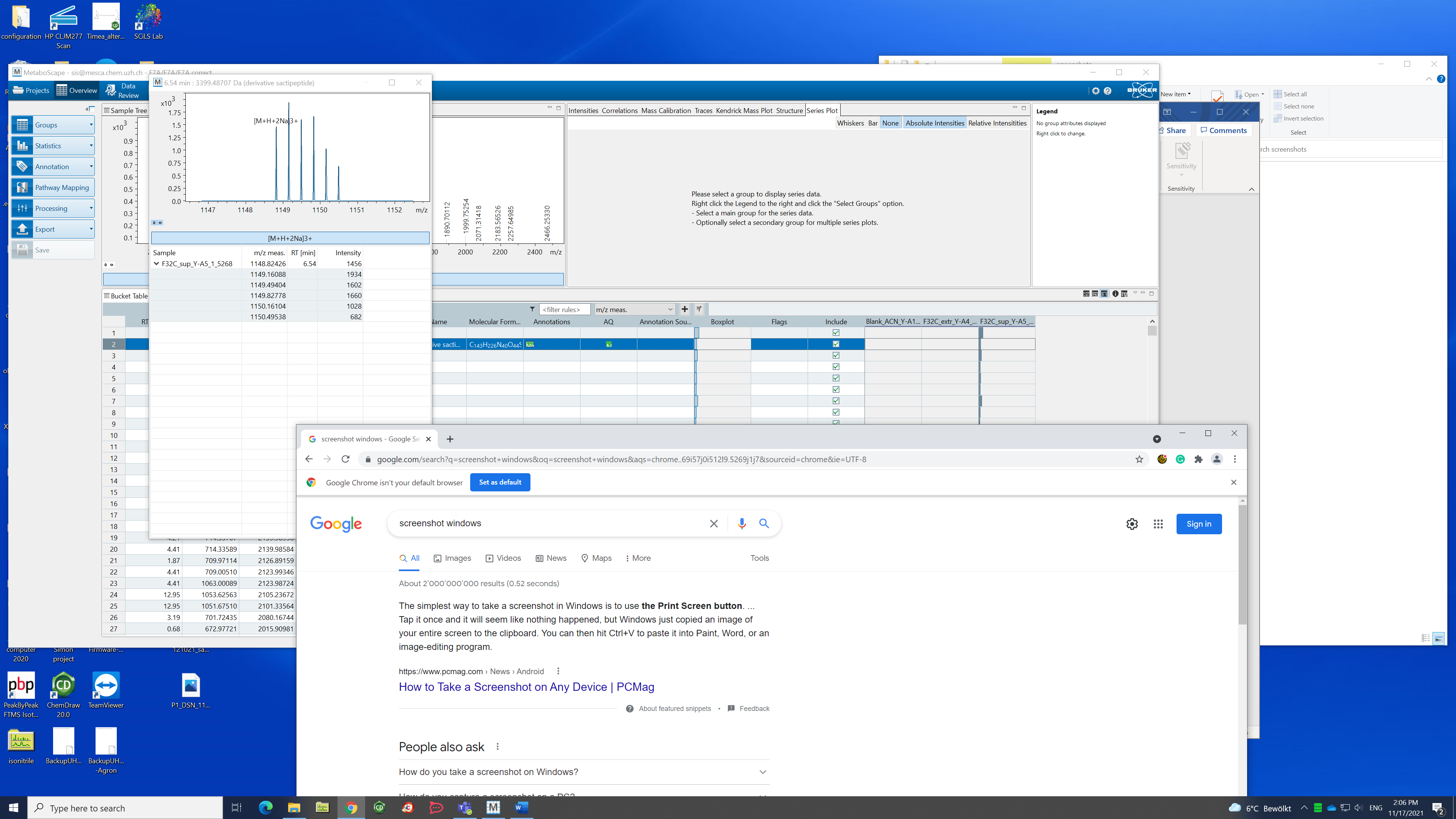


**Supplementary Figure 10.** HRMS of estercticin A detected in the supernatant of *C. estertheticum* CF004.
